# The FSHD Atrophic Myotube Phenotype Is Caused by DUX4 Expression

**DOI:** 10.1371/journal.pone.0026820

**Published:** 2011-10-28

**Authors:** Céline Vanderplanck, Eugénie Ansseau, Sébastien Charron, Nadia Stricwant, Alexandra Tassin, Dalila Laoudj-Chenivesse, Steve D. Wilton, Frédérique Coppée, Alexandra Belayew

**Affiliations:** 1 Laboratory of Molecular Biology, University of Mons, Mons, Belgium; 2 INSERM U1046, Le Centre Hospitalier Universitaire Arnaud de Villeneuve, Montpellier, France; 3 Molecular Genetic Therapy Group, University of Western Australia, Nedlands, Australia; Florida State University, United States of America

## Abstract

**Background:**

Facioscapulohumeral muscular dystrophy (FSHD) is linked to deletions in 4q35 within the *D4Z4* repeat array in which we identified the double homeobox 4 (*DUX4)* gene. We found stable DUX4 mRNAs only derived from the most distal *D4Z4* unit and unexpectedly extended to the flanking *pLAM* region that provided an intron and a polyadenylation signal. *DUX4* encodes a transcription factor expressed in FSHD but not control primary myoblasts or muscle biopsies. The DUX4 protein initiates a large transcription deregulation cascade leading to muscle atrophy and oxidative stress, which are FSHD key features.

**Methodology/Principal Findings:**

We now show that transfection of myoblasts with a *DUX4* expression vector leads to atrophic myotube formation associated with the induction of E3 ubiquitin ligases (MuRF1 and Atrogin1/MAFbx) typical of muscle atrophy. DUX4 induces expression of downstream targets deregulated in FSHD such as mu-crystallin and TP53. We developed specific siRNAs and antisense oligonucleotides (AOs) targeting the *DUX4* mRNA. Addition of these antisense agents to primary FSHD myoblast cultures suppressed DUX4 protein expression and affected expression of the above-mentioned markers.

**Conclusions/Significance:**

These results constitute a proof of concept for the development of therapeutic approaches for FSHD targeting *DUX4* expression.

## Introduction

Facioscapulohumeral muscular dystrophy (FSHD) is an autosomal dominant disorder affecting 1/17,000 births. It is characterised by muscle weakness and atrophy progressing from the face, the upper-arms and shoulder girdle to the lower limbs. FSHD1A (OMIM #158900) is genetically linked to contractions of the *D4Z4* repeat array in 4q35. Non-affected individuals typically present between 11–100 copies of the 3.3-kb *D4Z4* element in this locus while patients with FSHD only have 1–10 copies left [1]–[3]. A similar DNA hypomethylation associated with an open chromatin structure is observed both on contracted *D4Z4* arrays in FSHD1A and on normal-size arrays in FSHD1B (OMIM #158901) [4], [5].

**Figure 1:**
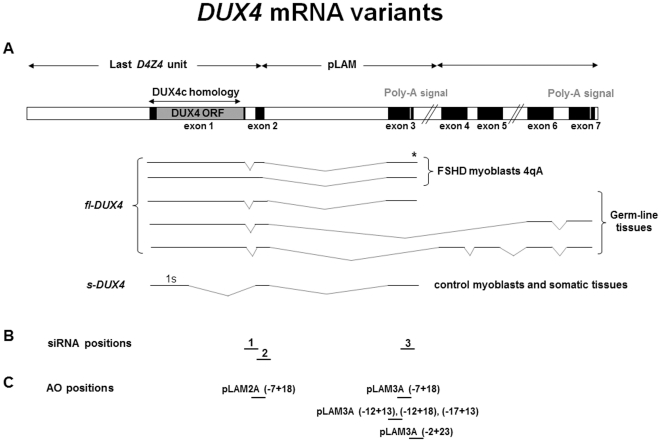
*DUX4* mRNA variants and positions of the siRNA and AOs target sequences. (**A**) (Top) Schematic representation of the last *D4Z4* unit, the adjacent *pLAM* region and the distal exons. The DUX4 ORF is contained in the first exon. The 5′UTR and a large part of the ORF identical to the DUX4c sequence is indicated. Two poly-A signals were reported [8], [11]. The *pLAM* region is only present on the 4qA allele and on the homologous chromosome 10 that has lost the poly-A signal. (Bottom) *DUX4* mRNA variants. All mRNAs reported to date in FSHD myoblasts containing the full ORF (*fl-DUX4*) end in exon 3. (*) *fl-DUX4* was also detected in control and FSHD fibroblast-derived iPS, in FSHD fibroblasts and in FSHD embryoid bodies. The shorter *s-DUX4* was detected in muscle and other somatic tissues [11]. These mRNAs derived exclusively from chromosome 4. The *fl-DUX4* was also detected in germ line tissue, some ending in exon 3 (4qA) and others in exon 7 derived from chromosome 4qA or the homologous 10qA. (**B**) Positions of the siRNA target sequences. (**C**) Positions of the AO target sequences.

The *D4Z4* unit contains a large open reading frame (ORF) with a double homeobox sequence [2] in which we mapped a functional promoter thus defining the *DUX4* gene [6], [7]. We could detect stable mRNAs comprising the full DUX4 ORF in FSHD but not control muscle cells. These *DUX4* mRNAs derived from the most distal unit, and unexpectedly extended within the flanking *pLAM* region that provided an intron and a polyadenylation signal (**Fig. 1A**, [8]). Investigations of genetic polymorphisms in a large cohort of patients and non-affected individuals confirmed this polyadenylation signal is needed to develop FSHD resulting in the production of stable *DUX4* mRNAs [9]. Other researchers could confirm the presence of *DUX4* mRNAs in FSHD muscle cells [9]–[11]. They further detected very low amounts of a short *DUX4* mRNA splice variant (*s-DUX4*) that encodes a non-toxic protein lacking the carboxyl-terminal domain in control muscles. A full lenght *DUX4* mRNA (*fl-DUX4)* was also characterized in induced pluripotent stem (iPS) cells and human testis, where the gene contains 4 additional exons and a more distal polyadenylation signal. Differentiation of iPS cells to embryoid bodies caused repression of this mRNA in control but not FSHD IPS cells [11].

The 52-kDa DUX4 protein is a potent transcription factor that may target numerous genes and its overexpression is toxic in cell cultures [12]–[14]. It directly activates the *PITX1* gene, which is specifically induced 10–15 fold in FSHD muscles as compared to 11 other neuromuscular disorders [8]. PITX1 is another homeodomain transcription factor [15]; its overexpression in skeletal muscles of a transgenic mouse caused reversible muscle atrophy [16]. *DUX4* overexpression in mouse *C2C12* cells recapitulated key features of the FSHD molecular phenotype, including repression of MyoD leading to differentiation defects, and repression of glutathione oxydo-reduction pathway components increasing sensitivity to oxidative stress [17]. Finally, *DUX4* overexpression in mouse muscles *in vivo* caused a TP53-dependent myopathy that required the DUX4 DNA binding domain [18]. *TP53* is a direct PITX1 target gene and thus belongs to the DUX4 transcription deregulation cascade [19]. In summary, these studies confirmed the major role played by DUX4 in the pathological mechanism of FSHD.

In addition, we have characterized the *DUX4c* (for centromeric) gene mapping 42 kb proximal of the *D4Z4* array. The encoded 47-kDa protein is identical to DUX4 except for the carboxyl-terminal region. DUX4c is expressed in control muscles, it is induced in muscles of patients affected with Duchenne muscular dystrophy and at similar or higher levels in FSHD muscles. DUX4c induced human myoblast proliferation, suggesting a role in muscle regeneration that might contribute to the FSHD pathology [20]. Additional genes, mapped in 4q35, were proposed to be activated in FSHD (*ANT1*, *FRG1*, *FRG2*) but several groups were unable to confirm these observations (reviewed in [3], [21]). Transgenic mice overexpressing one of these genes (*FRG1*) exhibited a form of muscular dystrophy [22].

In the present study, we identify FSHD markers associated with muscle atrophy that are induced by DUX4 expression and inhibited by its suppression either with short interfering RNAs (siRNAs) or antisense oligonucleotides (AOs). We present data establishing proof of concept in myoblast cultures that DUX4 inhibition can reverse the FSHD phenotype.

## Results

### DUX4 expression induces an atrophic myotube phenotype

**Figure 2:**
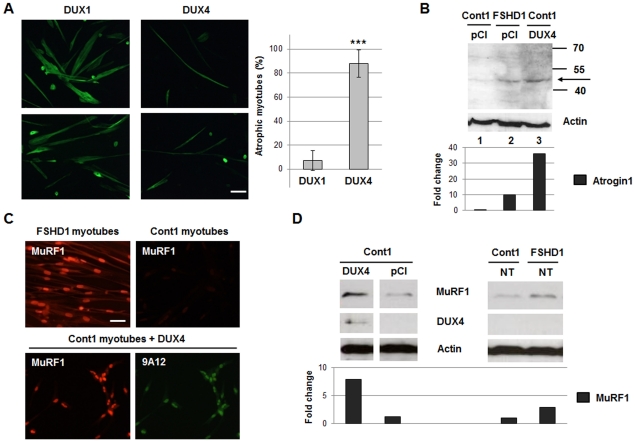
DUX4 overexpression induces muscle fiber atrophy and MuRF1 and Atrogin1 expressions. (**A**) Immortalized control myoblasts were transfected with *pCIneo-DUX1* (negative control) or *pCIneo-DUX4* expression vectors. Differentiation was induced 48 hours after transfection and 8 days later cells were fixed in 4% PAF and incubated with troponin T (myotube differentiation marker) antibody and a secondary antibody coupled to Alexa Fluor (green) (left). Scale bar: 15 µm. Means and SD of the ratio of atrophic versus total myotubes plotted (right, ***p<0.001). (**B–C**) Immortalised control myoblasts transfected with an expression vector for DUX4, and FSHD myoblasts were switched to differentiation medium. After 19 days a Western blot (B) and an immunofluorescence (C) were performed with an antibody against Atrogin1 and MuRF1, respectively. Actin was used as the loading control. Scale bar: 15 µm. (**D**) Immortalised control myoblasts transfected with the indicated expression vectors and FSHD myoblasts were switched to differentiation medium. After 15 days, a Western blot was performed with an antibody against MuRF1, appropriate secondary antibodies coupled to HRP and the Lumilight kit (Roche). 9A12 MAb staining confirmed DUX4 expression. The antibodies were then stripped, and the same membrane revealed with an anti-actin antibody to provide a loading control. A densitometry of the immunoreactive bands was performed. Data are normalized to actin levels in each sample.

In order to investigate whether DUX4 might interfere with the differentiation to myotubes, we transfected immortalized human control myoblasts with *pCIneo* vectors expressing DUX4 (**Fig. S1A**) or the shorter DUX1 protein, a non-4q35 homologue limited to the homeodomains [8]. We induced differentiation a few hours after transfection by a change in culture medium. In these conditions DUX4 doesn't exert its toxicity, and its expression can be observed in myotubes for several days [17, Tassin *et al*, 2011 in revision]. We detected troponin T, a cytoplasmic differentiation marker, by immunofluorescence 8 days after transfection. Most myotubes expressing DUX4 appeared much thinner with very limited amount of cytoplasm (**Fig. 2A**, right panels) than those expressing DUX1 (86 versus 8% of atrophic myotubes, p<0.001, **Fig. 2A**, left panels). This morphology was very similar to the previously described phenotype of atrophic FSHD myotubes [23].

Two muscle specific E3 ubiquitin ligases, Muscle ring finger 1 (MuRF1) and Atrogin1 (also named MAFbx), are upregulated prior to the onset of atrophy in multiple models of muscle wasting [24], [25]. Both proteins were induced in FSHD as compared to healthy control myotubes and detected by Western blot (**Fig. 2B and 2D**, right panel) and by immunofluorescence (**Fig. 2C**, upper panels). Both Atrogin1 and MuRF1 expression were induced in myotubes derived from control myoblasts transfected with *pCIneo-DUX4* as compared to the insertless *pCIneo* vector (**Fig. 2B**, lanes 1 and 3 **Fig. 2D**, left panel). MuRF1 co-localised with DUX4 in the nuclei of DUX4-expressing myotubes as detected by immunofluorescence (**Fig. 2C**, lower panel). These experiments show that DUX4 induces the expression of genes involved in muscle atrophy. The characteristic morphological changes induced by DUX4 expression in myotubes were thus considered as markers that would be useful in assessing inhibitory strategies against this protein.

### FSHD markers induced by DUX4 expression

**Figure 3:**
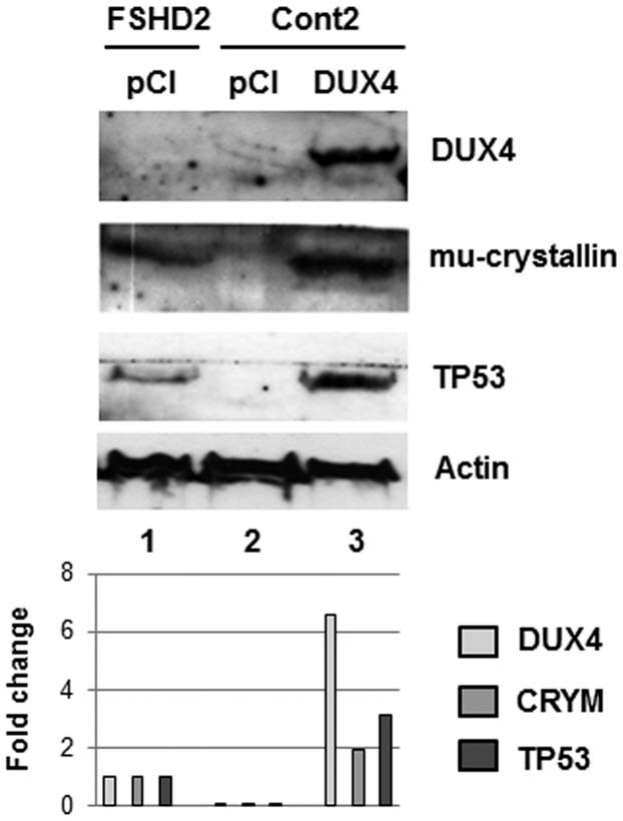
DUX4 protein overexpression induces different FSHD markers. 24 hours after seeding FSHD and control primary myoblasts were transfected with the indicated expression vectors. Total protein extracts were prepared 48 hours after transfection, 30 µg were separated by electrophoresis (12% PAGE-SDS), transferred to a Western blot and immunodetected with the indicated primary antibodies, appropriate secondary antibodies coupled to HRP and the Lumilight kit (Roche). Actin was stained by Ponceau red on the same membrane before immunodetection and was used as the loading control. A densitometry of the immunoreactive bands was performed. Data are normalized to actin levels in each sample.

We then analyzed the expression of different proteins known either to be induced in FSHD such as mu-crystallin (CRYM [26]), or to be induced by DUX4 such as TP53 [18]. A larger amount of these two proteins was observed upon immunodetection with specific antibodies on a Western blot prepared with total extracts of FSHD primary myoblasts as compared to control myoblasts (both cell types were transfected with the insert-less *pCIneo* vector; **Fig. 3**, lanes 1–2). In addition, these proteins were induced in control myoblasts upon transfection with the *pCIneo-DUX4* expression vector (**Fig. 3**, lane 3). DUX4 induced CRYM by direct promoter activation as shown by co-transfection with the DUX4 expression vector and a luciferase reporter gene fused to the *CRYM* promoter (**Fig. S1B**). The TP53 protein was similarly induced when myoblasts were transfected with a PITX1 expression vector (**Fig. S2A**) as previously shown in another cell type (MCF7 cells, [19]), indicating that TP53 was not directly induced by DUX4 but by activation of the *PITX1* gene.

In aggregate, these data suggested that Atrogin1, MuRF1, CRYM and TP53 could be considered as FSHD markers induced by DUX4 expression.

### Development of RNA interference against DUX4

We selected 3 *DUX4* mRNA sequences for siRNA targeting (Custom siRNA, Ambion) in the region most divergent from the highly similar *DUX4c* mRNA i.e. the 3′ untranslated region (3′UTR) transcribed from *pLAM* (**Fig. 1B**). We first transfected TE671 cells with these DUX4-siRNAs or a negative control siRNA (nc-siRNA), and then again 4 hours later with the *pCIneo-DUX4* expression vector that contains the full DUX4 ORF and the flanking *pLAM* region (**Fig. S1A**). siRNA transfection conditions are detailed in **Fig. S3** and **Table 1**. Cell extracts were prepared 1, 2 or 3 days after the second transfection and the DUX4 protein was immunodetected on Western blots (**Fig. S4A and S4B**). The DUX4- but not the nc-siRNAs strongly decreased DUX4 protein expression at 48 hours (**Fig. S4B**) and totally suppressed it at 72 hours (**Fig. S4A and S4B**). We selected siRNA3 for further studies as it mapped in the most DUX4-specific region. Because the *DUX4* and *DUX4c* mRNAs are highly similar, it was necessary to evaluate siRNA specificity. TE671 cells were transfected with a siRNA directed against the *DUX4* or *DUX4c* mRNA followed by transfection with the *pCIneo-DUX4* or *-DU*X4c expression vector. The siRNA specificity was shown by the disappearance, in Western blot, of the immunodetected bands corresponding to either the DUX4 or DUX4c protein following the addition of their respective siRNA but not the siRNA of their homologue (**Fig. S4C**).

**Table 1:** Transfection conditions.

Cell lines	Transfection reagents	Transfection efficiency	Differentiation induction	Figures
**Human Immortalized** **Myoblasts**	NanoJuice (Novagen):expression vectors	80% at 48 hours	48 hours after transfection	S7A, B
**Human Primary Myoblasts**	Fugene HD (Roche):expression vectorsand AOs	80% at 48 hours	4 hours after transfection	S7C, D
	siPORTNeoFX (Ambion):siRNA	DUX4 suppressionat 72 hours	4 hours after transfection	5A
**TE671** (Human RhabdomyosarcomaAlveolar cells)	Fugene 6 (Roche):expression vectors	80–90% at 24 hours		S4B
	siPORTNeoFX (Ambion):siRNA	DUX4 suppressionat 72 hours		S4B
**C2C12**(Mouse myoblasts)	Lipofectamin 2000 (Invitrogen):expression vectors	80–90% andDUX4 suppressionat 24 hours		S5

### RNA interference against DUX4 prevents development of the atrophic myotube phenotype

**Figure 4:**
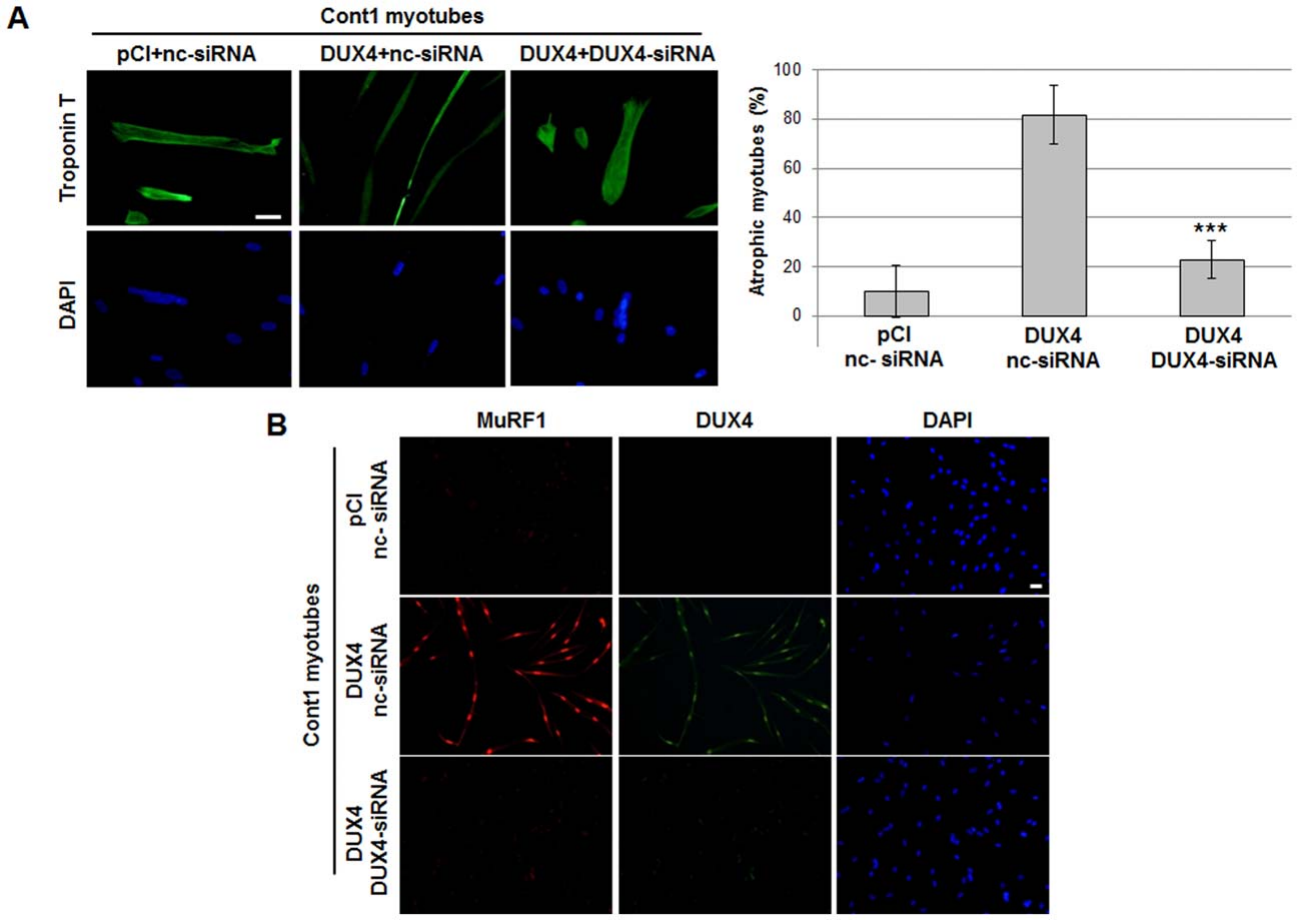
RNA interference against DUX4 reverts the atrophic myotube phenotype and decreases MuRF1 expression. (**A**) Control immortalised myoblasts were transfected with a negative control siRNA (nc-siRNA) or DUX4-siRNA (10 nM) using reverse transfection and transfected again 4 hours later with the *pCIneo* or *pCIneo-DUX4* (DUX4) expression vector. The 3rd day after *pCIneo* vector transfection, cell differentiation was induced. Eight days later cells were fixed in 4% PAF and incubated with troponin T antibody and a secondary antibody coupled to Alexa Fluor (green). The nuclei were labeled with DAPI. Scale bar: 15 µm. Means and SD of the ratio of atrophic versus total myotubes was performed and plotted (right, ***p<0.001). (**B**) Control immortalised myoblasts were transfected and differentiated as described above. Eight days later cells were fixed in 4% PAF and incubated with troponin T (green) or MuRF1 (red) primary antibodies and appropriate secondary antibody (Alexa Fluor). The nuclei were labeled with DAPI. Scale bar: 15 µm.

We then investigated whether the DUX4-siRNA could prevent formation of atrophic myotubes. We transfected immortalised control myoblasts with both the *pCIneo-DUX4* expression vector and the DUX4-siRNA as above, induced differentiation and examined the myotube morphology 8 days later. Immunofluorescent staining for troponin T (green) demonstrated that DUX4-expressing myotubes treated with the nc-siRNA appeared much thinner than those treated with the DUX4-siRNA (82 versus 9% of atrophic myotubes, p<0.001, **Fig. 4A**, right panels). Immunofluorescent staining for MuRF1 (red), an atrophy marker that colocalized with DUX4 (green) in nuclei was also decreased in control myotubes transfected with *pCIneo-DUX4* and the DUX4-siRNA (**Fig. 4B**, lower panels) as compared to the use of a nc-siRNA (middle panels).

### RNA interference against endogenous DUX4 in FSHD primary myotubes

**Figure 5:**
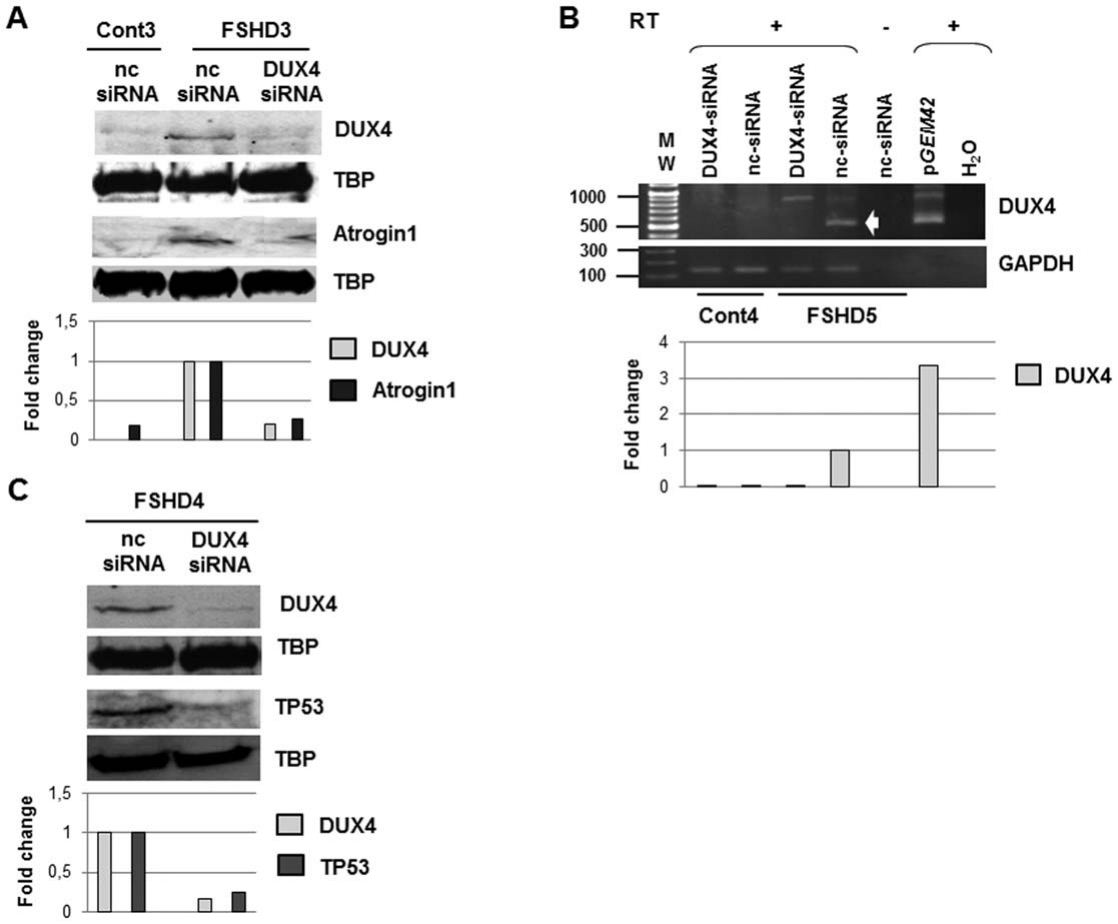
Evaluation of DUX4-siRNA efficiency on endogenous DUX4 and FSHD marker expression in FSHD primary myoblasts. (**A**) 10^5^ cells were seeded in 35 mm culture dish and directly transfected with negative control siRNA (nc-siRNA, 30 nM) or DUX4-siRNA3 (10 nM) using the reverse transfection method with 4 µl of siPORTNeoFX reagent. Differentiation was induced 4 hours after transfection, and cells were harvested 72 hours later. A nuclear extract was prepared and 20 µg of nuclear proteins were separated in parallel by two electrophoresis (12% PAGE-SDS), and transferred onto a nitrocellulose membrane. The proteins transfer was confirmed by Ponceau red staining. After rinsing the membranes were incubated either with 9A12 MAb or a polyclonal antibody against Atrogin1 (ECM Biosciences) followed by secondary antibodies coupled to horseradish peroxidase and revealed with the Femto Super Signal kit (Pierce). The antibodies were then stripped, and the same membranes revealed with an anti-TBP MAb (nuclear loading control). (**B**) Primary FSHD and control myoblasts transfected with the DUX4-siRNA (10 nM) or the negative control siRNA (nc-siRNA, 30 nM) were differentiated for 3 days. Total RNA was extracted. Reverse transcription was performed on 500 ng of DNase-treated total RNA with the 3′adaptator of the RLM-RACE kit (Ambion). 5 µl of the resulting cDNA were amplified by nested PCR (for details, see methods). The RT-PCR products were analysed by electrophoresis on an 1% agarose gel. A densitometry of the bands was performed for quantification. Data are normalized to GAPDH levels in each sample. *pGEM42*: expression vector containing 2 *D4Z4* units (7); RT (+): with reverse transcriptase; (−): without reverse transcriptase. H_2_O: RT-PCR was performed with H_2_O. GAPDH: internal control. (**C**) Immunodetection of either DUX4 or TP53 with specific primary antibodies and appropriate secondary antibodies as described in the legend to **Fig. 3** on two Western blots prepared with nuclear extracts of myotubes as described in **Fig. 5A**. A densitometry of the immunoreactive bands was performed. Data are normalized to TBP levels in each sample.

We determined the optimal transfection conditions of human primary myoblasts with the siRNA against GAPDH as above (**Fig. S3B**, **Table 1**). In these conditions, we transfected FSHD primary myoblasts with 10 nM DUX4-siRNA and induced differentiation 4 hours later, since the endogenous DUX4 protein is more easily detectable in myotubes than in myoblasts (Tassin *et al*, 2011 in revision). Three days later, nuclear extracts were analysed by Western blot: a significant decrease of the immunodetected DUX4 protein amount was observed (**Fig. 5A**, upper panel) as compared to cells treated with the nc-siRNA. We also investigated Atrogin1 expression (**Fig. 5A**): a band was immunodetected in nuclear extracts of FSHD myotubes treated with the nc-siRNA and disappeared upon treatment with DUX4-siRNA. This was not caused by a general decrease in nuclear protein expression since the amounts of TBP (TATA binding protein) were unchanged (**Fig. 5A**, lower panel). A reverse transcription (RT) and amplification by PCR with primers previously shown to be specific of the *DUX4* mRNA 3′UTR [8] were carried out on myotube total RNA. The expected 550 bp DNA fragment was detected in FSHD myotubes treated with the nc-siRNA and at a 80% reduced intensity in cells treated with the DUX4-siRNA (**Fig. 5B**). This amplicon was observed in the positive control i.e. C2C12 cells transfected with the *pGEM42* vector containing two *D4Z4* units [7] but not in primary myoblasts from a healthy donor, or upon omission of reverse transcriptase. Products were cloned and sequenced to confirm *DUX4* mRNA amplification (data not shown). The RT-PCR product of *GAPDH* mRNA amplification was used as an internal control.

### RNA interference against endogenous DUX4 suppresses expression of FSHD markers

To test the efficacy of the DUX4-siRNA, we then investigated the expression of two markers that are induced in FSHD as well as following the transcription deregulation cascade initiated by DUX4. We selected TP53 that is activated by PITX1, itself activated by DUX4 (**Fig. 3B** **and** **S2A**; [8], [18]). We used the same experimental protocol as in **Fig. 5A**. A strong decrease in the amount of TP53 was observed by immunostaining on a Western blot prepared with lysates of cells treated with DUX4-siRNA as compared to cells treated with the nc-siRNA (**Fig. 5C**).

### Development of splice switching antisense oligonucleotides to downregulate DUX4

RNA-like antisense oligonucleotides (AOs) are being used in a therapeutic approach for Duchenne muscular dystrophy. The antisense oligomer induces removal of an exon flanking a frame-shifting exonic deletion from the dystrophin gene transcript and restores the reading frame, allowing synthesis of a semi-functional dystrophin isoform [27]. Inversely when an exon is targeted for removal from a normal dystrophin gene transcript, the reading-frame may be disrupted and this resulted in a transient phenocopy of gene inactivation [28]. We thus wanted to similarly develop specific AOs interfering with *DUX4* mRNA processing and/or stability. We designed 2′-O-methyl modified bases on a phosphorothioate backbone complementary to regions in the *DUX4* gene sequence we had characterized (GenBank # AF117653), and targeted acceptor splice sites of *pLAM* exons 2 and 3 involved in pre-mRNA splicing (**Fig. 1C**). The splice-switching efficacy of these 25–30 mer AOs was first evaluated by co-transfection of C2C12 mouse myoblasts, as previously described for Duchenne AOs [29]. The cells were lysed 24 hours after transfection with the *pCIneo-DUX4* expression vector and analysed by Western blot as above: no DUX4 protein was immunodetected following the addition of the 600 nM AOs directed against the *DUX4* pre-mRNA (data not shown). In contrast DUX4 was clearly expressed in cells treated with AO mGMCSF3A(−5+20), an unrelated negative control AO (nc-AO) targeting the murine GMCSF pre-mRNA or in the absence of AO (data not shown). However, a specificity problem was observed: when cells were co-transfected with the *pCIneo-DUX4c* expression vector and AOs directed against DUX4, expression of the homologous DUX4c protein was also decreased (data not shown). The high AO concentration used (600 nM) in these experiments most probably explain this result, as we have previously observed mismatched AOs can induce some exon skipping when applied at high concentrations [30].

### Determination of specific concentrations for AOs against DUX4

We then defined the minimal AO concentrations allowing DUX4 inhibition without affecting DUX4c protein levels using the same transient expression approach as above. We then evaluated different AO concentrations in C2C12 cells co-transfected with *pCIneo-DUX4* and *-DUX4c*, so that both mRNAs were present simultaneously in the same cells. In these conditions, a 150 nM concentration appeared best since it nearly suppressed the DUX4 protein but only had a minimal influence on DUX4c (data not shown). We tested several other AOs directed against DUX4 at this concentration in co-transfected C2C12 cells. In these conditions, AOs pLAM3A(−2+23), pLAM3A(−12+13) and pLAM3A(−7+18) could strongly reduce DUX4 protein levels as compared to the nc-AO, while DUX4c was still expressed (**Fig. S5A**). The optimal AO concentration was respectively 50 nM for pLAM2A(−7+18) (**Fig. S5B**) and 10 nM for pLAM3A(−12+18) and pLAM3A(−17+13) (**Fig. S5C**).

### Antisense oligonucleotides suppress endogenous DUX4 expression in FSHD primary myotubes

**Figure 6:**
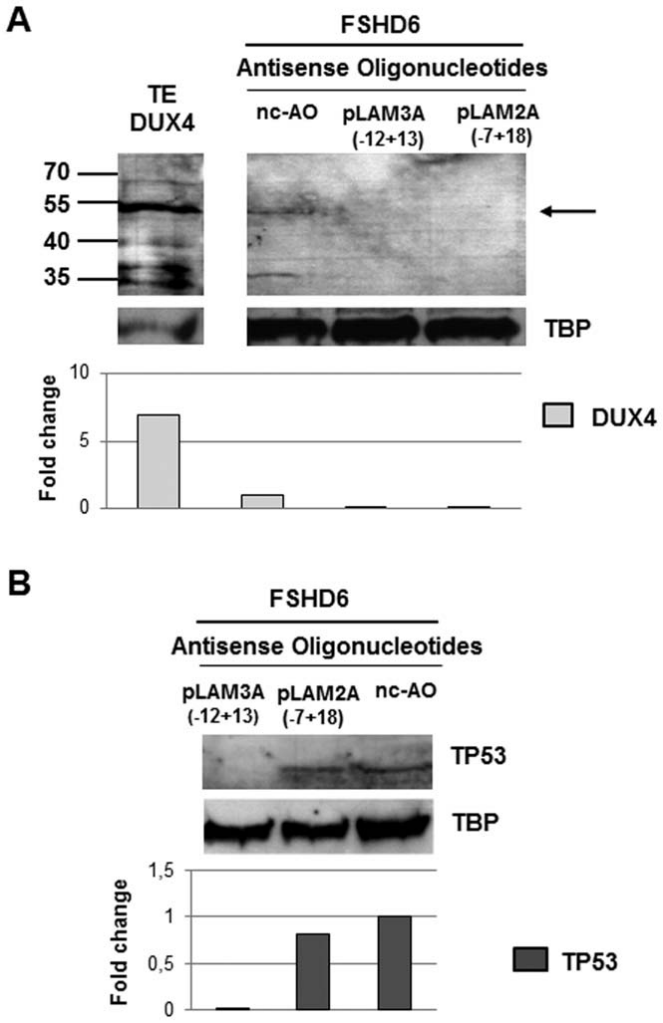
Efficiency of AOs pLAM2A (−7+18) and pLAM3A (−12+13) in suppressing endogenous DUX4 and TP53 expression in primary FSHD myotubes. (**A**) 10^5^ primary FSHD myoblasts were seeded in 35 mm culture dishes. The next day, cells were transfected with either the negative control AO mGMCSF3A(−5+20) (nc-AO, 600 nM) or AOs pLAM2A (−7+18) (50 nM) or pLAM3A (−12+13) (150 nM). Differentiation was induced 4 hours after transfection and cells were harvested 72 hours later. Nuclear extracts were prepared and 20 µg of proteins were separated by electrophoresis (12% PAGE-SDS), transferred to a Western blot and DUX4 was immunodetected with 9A12 MAb. The antibodies were then stripped, and the same membrane used for immunodetection of TBP. The methodology for the Western blot is shown in the legend to **Fig. 5**. TE-DUX4: positive control, 5 µg protein extract of TE671 cells transfected with a *pCIneo-DUX4* expression vector (**B**) Immunodetection of TP53 with specific primary antibody and appropriate secondary antibody as described in the legend to **Fig. 3** on Western blot prepared with protein extracts of cells used in the above experiment (**6A**). A densitometry of the immunoreactive bands was performed. Data are normalized to TBP levels in each sample.

**Figure 7:**
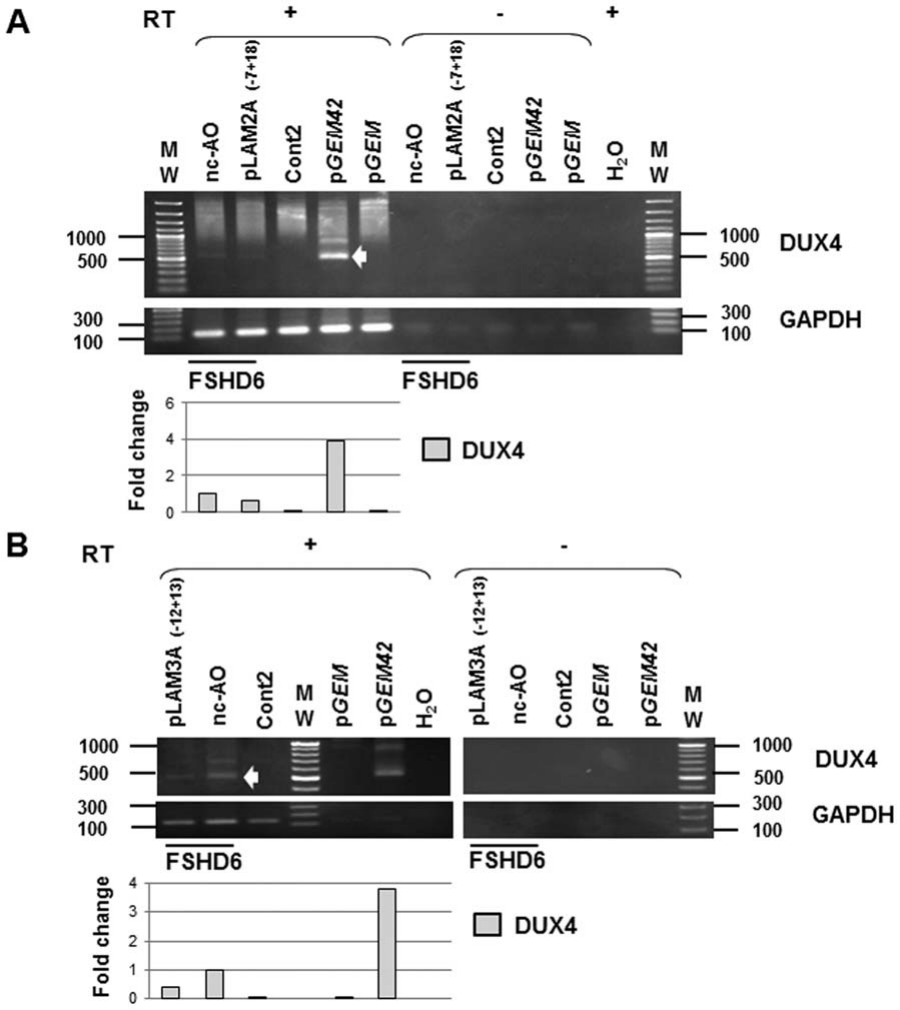
Efficiency of AOs pLAM2A (−7+18) and pLAM3A (−12+13) in suppressing endogenous DUX4. Primary FSHD myoblasts transfected with AOs pLAM2A (−7+18) (50 nM) (**A**) or pLAM3A (−12+13) (150 nM) (**B**) or the negative control AO mGMCSF3A(−5+20) (nc-AO, 600 nM) were differentiated for 3 days. Total RNA was extracted. RT-PCR was performed as described in **Fig. 5B**. The RT-PCR products were analysed by electrophoresis on 1% agarose gel. A densitometry of the bands was performed for quantification. Data are normalized to GAPDH levels in each sample. *pGEM42*: expression vector containing 2 *D4Z4* units [7]; *pGEM:* empty expression vector; RT (+): with reverse transcriptase; (−): without reverse transcriptase. H_2_O: RT-PCR was performed with H_2_O. GAPDH: internal control.

To test the efficacy of AOs pLAM2A(−7+18) and pLAM3A(−12+13) on endogenous DUX4 expression, we transfected primary FSHD myoblasts with the optimal concentrations defined above. Differentiation was induced 4 hours after transfection and three days later myotubes were lysed for either protein analysis or total RNA extraction. The DUX4 protein was immunodetected on Western blot in lysates of cells treated with the nc-AO but not anymore in those treated with AOs pLAM2A(−7+18) and pLAM3A(−12+13) (**Fig. 6A**). An RT-PCR was carried out on myotube total RNA as described in **Fig. 5B**. The expected 550 bp DNA fragment was detected in FSHD myotubes treated with nc-AO and at a 30% and 50% reduced intensity in cells treated with AOs pLAM2A(−7+18) (**Fig. 7A**) or pLAM3A(−12+13) (**Fig. 7B**), respectively. This amplicon was observed in the positive control i.e. C2C12 cells transfected with the *pGEM42* but not in the negative controls i.e. either C2C12 cells transfected with the empty *pGEM* vector, or primary myoblasts from a healthy donor, or upon omission of reverse transcriptase. Products were cloned and sequenced to confirm *DUX4* mRNA amplification (data not shown). The RT-PCR product of *GAPDH* mRNA amplification was used as an internal control.

### Antisense oligonucleotides against endogenous DUX4 suppress FSHD markers expression

To test the efficacy of AOs against DUX4, we investigated as previously the expression of TP53. We used the same protein extract as in **Fig. 6A**, and a decrease in the amount of TP53 was observed on Western blot in lysates of cells treated with AOs pLAM2A(−7+18) and pLAM3A(−12+13) as compared to cells treated with the nc-AO (**Fig. 6B**). The lower decrease with the first AO targeting the alternative by spliced intron I is in concordance with the low reduction in *DUX4* mRNA evidenced by RT-PCR (**Fig. 7**). In contrast the AO targeting intron II that is always spliced out leads to an undetectable TP53 level [8], [10]. This experiment confirmed that DUX4 suppression affected a gene downstream in the gene deregulation cascade it induced.

## Discussion

### DUX4 activates the muscle atrophy pathway in myoblast cultures

In the present study, we have shown that DUX4 overexpression in human primary myotubes induced Atrogin1 (MAFbx) and MuRF1 activation, two genes specific of the muscle atrophy pathway. They encode E3 ubiquitin ligases that bind to myofibril proteins, cause their ubiquitination and subsequent degradation via the proteasome [25]. Accordingly, myotubes expressing *pCIneo-DUX4* were much thinner than myotubes containing an insertless control vector and similar to the phenotype of atrophied FSHD myotubes described in [23]. This atrophic phenotype as well as Atrogin1 and MuRF1 activation could be reverted by RNA interference against DUX4, further demonstrating its role in the FSHD pathological process. We thus propose Atrogin1 and MuRF1 as FSHD markers, although it is not clear whether the Atrogin1 and MuRF1 genes are direct DUX4 transcriptional targets or are further down in the activation cascade. Indeed a putative PITX1 binding site has been found in the Atrogin1/MAFbx promoter, and it was shown that PITX1 overexpression in skeletal muscles induced atrophy in a mouse transgenic model [16].

### Additional FSHD markers

We have shown that *DUX4* overexpression could activate other markers induced in FSHD such as mu-crystallin (CRYM) or TP53. Reed *et al.* have reported that mu-crystallin (CRYM) protein levels were up-regulated in FSHD deltoid muscles but not in several other myopathies [26]. Klooster *et al.* could not confirm this FSHD-specific up-regulation in quadriceps biopsies, and also found high *CRYM* mRNA and protein expression levels in some normal control samples [31]. This might reflect a muscle type specificity in CRYM induction. CRYM is a thyroid-hormone binding protein with a NADPH-dependent activity and so influences differentiation and oxidative stress responses [32] reported to be altered in FSHD [33]–[35]. A recent study has shown that overexpression of p43, a T3 thyroid-hormone mitochondrial receptor, could induce skeletal muscle atrophy with an increase of oxidative stress. This muscle atrophy was caused by induction of the ubiquitin proteasome pathway involving Atrogin1 and MuRF1 [36]. CRYM is also linked to retinal and inner ear defects, common in FSHD, suggesting that its up-regulation might play a role in the disease pathogenesis [37]–[40].

**Figure 8:**
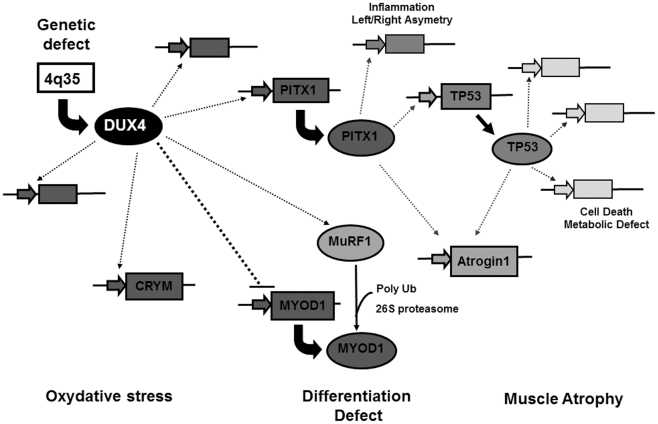
Schematic representation of the transcriptional cascade induced by mis expression of DUX4 in FSHD. The *DUX4* gene mapped in the *D4Z4* repeated element at 4q35 encodes a transcription factor that can directly interact with a set of target genes. Among those DUX4 inhibits the *MYOD1* gene that encodes the transcription master switch of muscle differentiation thus causing inhibition of the MYOD1 target genes in FSHD. DUX4 also inhibits the expression of genes involved in response to oxidative stress, and induces the mu-crystallin (*CRYM*) gene. Another direct DUX4 target gene is *PITX1* specifically induced in FSHD muscles as compared to 11 neuromuscular disorders; it induces E3 ubiquitin ligases (Atrogin1 and MuRF1) linked to atrophy in adult skeletal muscles and is involved in inflammation. The MuRF1 protein causes a.o. MYOD1 polyubiquination and proteasome-mediated degradation. Among the PITX1 target genes is *TP53* that has major roles in the control of DNA repair, cell cycling and apoptosis as well as at multiple levels of cell metabolism. Legend: Activate: 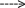 Inhibit: -----.

The tumor suppressor TP53 is a transcription factor that negatively regulates cell proliferation and survival. Its expression is maintained at a very low level during normal cell growth through regulation by proteosomal degradation [41]. However, the TP53 protein is both stabilized and activated in response to DNA damage, oncogene activation, hypoxia, nutrient deprivation and other stress-related signals. TP53 is also an important regulator of metabolic pathways. By transcriptional activation and other means, TP53 can contribute a.o. to the regulation of glycolysis, oxidative phosphorylation, fatty acid oxidation, oxidative stress and antioxidant response, mitochondrial integrity, autophagy and mTOR signaling [42]. A link between TP53 and the DUX4-mediated myopathy was established by Wallace *et al*, as TP53 inhibition mitigated *DUX4* toxicity *in vitro*, and muscles from TP53 null mice were resistant to *DUX4*-induced damage [18]. The PITX1 transcription factor directly activated transcription of the TP53 gene in MCF-7 mammary carcinoma cells resulting in cell-cycle arrest and TP53-dependent apoptosis [19]. We showed here that PITX1 could also induce TP53 in human primary myoblasts. Since DUX4 directly activates transcription of the PITX1 gene in myoblasts, TP53 can be considered an FSHD marker as part of the gene deregulation cascade initiated by DUX4 (**Fig. 8**, [8]).

### Therapeutic approaches

No therapeutic strategies targeting the FSHD molecular cause has been described to-date. Because of the pivotal role caused by DUX4 expression in the FSHD pathology [8], [9], [17], [18] we wished to suppress its expression using small double-stranded RNAs (siRNAs) or antisense oligonucleotides (AOs) in the aim to develop therapeutic strategies for FSHD. AOs can redirect gene expression through RNA silencing [43], suppressing specific mRNA translation [44], [45], altering mRNA stability [46], and/or redirecting pre-mRNA splicing patterns to disrupt the mature mRNA [27], [28]. We have thus focused two distinct mechanisms of antisense gene silencing or splice-switching technologies to block DUX4 protein expression. As the *DUX4* transcript is very similar to the homologous *DUX4c* mRNA, we targeted the most divergent region, located in the *DUX4* 3′UTR. We could demonstrate the specificity of these antisense agents since at lower concentrations they could mediate DUX4 suppression without interfering with the expression of the homologous DUX4c protein. As the endogenous DUX4 protein is present at high level in very few FSHD primary myotubes, it was appropriate to select the lower antisense agent concentration showing inhibition in cells transfected with the strong *pCIneo-DUX4* expression vector. This concentration appears sufficient to suppress the endogenous DUX4 protein. Since the DUX4 protein expressed in FSHD is only detectable at the myotube stage, it was necessary to establish transient transfection procedures for cultured myoblasts in which the siRNA or AO enters the cells with high efficiency and without significant cytotoxicity. Under these conditions, differentiation can be induced a few hours after transfection and myotubes harvested 3 days later for analysis.

We have identified several useful antisense agents targeting the *DUX4* pre-mRNA, preventing DUX4 protein expression and affecting the FSHD markers defined above as part of the DUX4 deregulation cascade. Among these the amount of TP53 protein appeared decreased in relation with the residual amounts of *DUX4* mRNA detected by RT-PCR (80% with siRNA and 30% or 50% with AOs pLAM2A(−7+18) or pLAM3A(−12+13), respectively). These antisense agents target both *DUX4* transcript variants: (i) the full-length *DUX4* mRNA (*fl-DUX4*) produced from the last *D4Z4* unit in FSHD and extended to the flanking *pLAM* region that provides a polyadenylation signal [8], [11]; (ii) the shorter *DUX4* mRNA (*s-DUX4*) that ends at the same *pLAM* polyadenylation site and uses a cryptic splice donor site within the DUX4 ORF that would limit a putative expressed protein to its double homeodomain [11]. The *fl-DUX4* mRNA is only detected in FSHD muscle cells and biopsies, whereas *s-DUX4* is detected both in control and some FSHD samples [11]. It is possible that any antisense strategies affecting the *s-DUX4* mRNA could be detrimental to control cells, but *s-DUX4* mRNA degradation does not seem to be problematic since healthy homozygous 4qB/4qB individuals were described who do not produce this transcript isoform [47]. Moreover the *s-DUX4* mRNA was only mentioned in one report [10] but not in the second [11] of the same group suggesting it is not present in every individual.

In conclusion we have demonstrated a biological impact of DUX4 inhibition leading to decreased atrophy markers and phenotype observed in FSHD and resulting from DUX4 expression. These diverse strategies seem promising and could contribute to future development of therapeutic approaches for FSHD as well as confirming the important role of DUX4 in the pathogenesis of this disease.

## Materials and Methods

### Ethics Statement

Primary human myoblasts were derived from muscle biopsies performed according to current ethical and legislative rules of France and written informed consent was obtained from all subjects, as directed by the ethical committee of CHU de Villeneuve (Montpellier, France) [23]. In addition, the uses of this material have been approved by the ethics committee of the University of Mons (ref # A901).

### Myogenic cell cultures and DNA vector transfection

C2C12 (mouse myoblast) and TE671 (human rhabdomyosarcoma) cells were grown in DMEM High Glucose (4.5 g/l) with L-Glutamine and Sodium pyruvate (PAA Laboratories GmbH, Pasching, Austria), 1% Antibiotic/antimycotic (PAA Laboratories GmbH) and 10% Fetal Bovine Serum Gold (PAA Laboratories GmbH) at 37°C under 5% CO_2_. For transfection, C2C12 cells were seeded in 6-well plates (Greiner bio-one, Frickenhausen, Germany) and transfected 24 hours later in Opti-MEM (Invitrogen, CA, USA) with Lipofectamin 2000 (µl) and DNA vector (µg) at a 10∶2 ratio according to the manufacturer's instructions (Invitrogen). TE671 cells were transfected in culture medium, 24 hours after seeding, with Fugene 6 (Roche Diagnostics GmbH, Mannheim, Germany) and DNA vector at a 4∶1 ratio according to manufacturer.

Immortalized human myoblasts have been kindly provided by Drs. G. Butler-Browne and V. Mouly (Institute of Myology, Paris). These lines were derived from primary myoblasts of a patient with FSHD (2 *D4Z4* units; FSHcl17) and a non-affected control (LHCN-M2); they were immortalized as described in [48], **Table 2**. The endogenous DUX4 protein was detected by Western blot on nuclear extracts of FSHD but not control immortalized myotubes (**Fig. S6A**). Myoblasts were grown in DMEM with 4.5 g/l Glucose and L-Glutamine (Lonza, Verviers, Belgium) with 20% 199 medium (Invitrogen), Gentamicin (50 µg/ml, Sigma-Aldrich, St Louis, USA), 20% Fetal Bovine Serum (Invitrogen), HGF (1 µg/ml, Sigma-Aldrich) and dexamethazone (20 µg/ml, Sigma-Aldrich) at 37°C under 5% CO_2_. Confluent myoblast cultures were differentiated by replacing the medium to DMEM/Gentamicin (50 µg/ml) without serum but supplemented with insulin (10 µg/ml, Sigma-Aldrich) and apotransferrin (100 µg/ml, Sigma-Aldrich) during 3–5 days. For transfection, myoblasts were transfected in culture medium, 24 hours after seeding, with NanoJuice (Novagen, WI, USA) and DNA vector at 1∶1 ratio according to the manufacturer. The transfection efficiency was at its maximum after 48 hours (**Fig. S7A and S7B**).

**Table 2:** Data of patients with FSHD and control individuals.

Code	References	Age	Sex	*D4Z4* units	Figures
**FSHD1**	Immortal myoblasts (FSHDcl17)Institute of Myology, Paris	27	M	2	2, 4, S6A
**Cont1**	Immortal myoblasts (LHCN-M2)Institute of Myology, Paris	41	M	>10	2, 4, S6A
**FSHD2**	Primary myoblasts described inBarro *et al*, 2008 (FSHD14)	25	M	4	3, S6B
**FSHD3**	Primary myoblasts described inBarro *et al*, 2008 (FSHD8)	39	M	6	5A
**FSHD4**	Primary myoblasts described inBarro *et al*, 2008 (FSHD7)	53	M	9	5C, S6C
**FSHD5**	Primary myoblasts described inBarro *et al*, 2008	46	M	5	5B
**FSHD6**	Primary myoblasts described inBarro *et al*, 2008 (FSHD5)	53	M	6	6, 7
**FSHD7**	Primary myoblasts described inBarro *et al*, 2008 (FSHD10)	20	F	4	S6C
**Cont2**	Primary myoblasts described inBarro *et al*, 2008 (ctl10)	21	M	>10	3, 7
**Cont3**	Primary myoblasts described inBarro *et al*, 2008 (ctl14)	43	M	>10	5A
**Cont4**	Primary myoblasts described inBarro *et al*, 2008 (ctl9)	24	F	>10	5B, S6B
**Cont5**	Primary myoblasts described inBarro *et al*, 2008 (ctl3)	41	M	>10	S6C

Primary human myoblasts from an unaffected control and a patient with FSHD were isolated from muscle biopsies, purified and established as described ([23], **Table 2**). The endogenous DUX4 protein was detected by Western blot in nuclear extracts of each FSHD but not control primary myotube cultures (**Figs. 5** **,** **6** **, S6B and S6C**). They were grown in 35 mm collagen-coated dishes (Ywaki, Japan) in DMEM with 4.5 g/l Glucose and L-Glutamine (Lonza) with Gentamycin (50 µg/ml, Sigma-Aldrich), 10% Fetal Bovine Serum (Invitrogen), and 1% Ultroser G (Pall BioSepra, Cergy-St-Christophe, France) at 37°C under 5% CO_2_. Confluent myoblast cultures were differentiated by replacing the medium to DMEM/Gentamicin (50 µg/ml) with 2% FBS during 3–5 days. Myoblasts were transfected in their culture medium, 24 hours after seeding, with Fugene HD (Roche Diagnostics) and DNA at a 6∶2 ratio according to the manufacturer (**Fig. S7C and S7D**).

### siRNA design and transfection

Short interfering (si)RNAs were designed using the Eurogentec siRNA Design Service on the *DUX4* 3′UTR and *in vitro* synthesized with the siRNA Construction Kit (Applied Biosystems Ambion, Austin, Texas (**Fig. 1**). For cell transfection, we used the “Silencer siRNA Starter Kit” (Applied Biosystems Ambion) with the “siPORTNeoFX” transfection agent. This kit also contains two control siRNAs: a GAPDH-siRNA and a negative control siRNA (nc-siRNA), with no significant similarity with transcribed sequences of human, mouse or rat. We used “reverse” transfection in which the reagent is introduced into the culture dishes before seeding the cells. We used 2 µl siPORTNeoFX and 10 nM siRNA for TE671 transfection or 4 µl siPORTNeoFX and 10 nM siRNA for primary myoblasts according to the manufacturer. All transfections were done in duplicate wells and repeated 3 times to ensure consistency.

### AOs design, synthesis and transfection

We used 25–30 mer *2′-O-*methyl phosphorothioate oligonucleotides (AOs) (positions given in **Fig. 1C**) produced from the sequence of the *DUX4* gene we characterized (Genbank # AY044051.4). They were synthesized on an Expedite 8909 Nucleic Acid Synthesizer using the 1 micromole Thioate protocol at the ANRI (Australian Neuromuscular Research Institute, Nedlands, WA, Australia) [27]. Splice switching AO nomenclature is based upon that described by Mann *et al.* [49]. The first letters designate the region (here, *pLAM* region), the number indicates the exon, the second letter specifies Acceptors or Donor splice sites, with the +/− and numbers representing the annealing coordinates in the intronic and exonic domain respectively. For example, pLAM3A(−12+13) will target acceptor site for exon 3, last 12 bases of intron II and 13 bases of exon 3.

C2C12 cells were transfected 24 hours after seeding in Opti-MEM medium (Invitrogen) using Lipofectamin 2000 and different AO ratios as indicated in the figure legends. For each experiment, transfections were repeated 3 times to confirm reproducibility. Primary human myoblasts were transfected in their culture medium, 24 hours after seeding, with Fugene HD (Roche Diagnostics) and different AO ratios as indicated in the figure legends. All transfections occurred in duplicate wells and were repeated 3 times to ensure consistency.

### Immunofluorescence staining

TE671 or human myoblasts were fixed in PBS containing 4% paraformaldehyde (Sigma-Aldrich) and treated with PBS 0.5% Triton X-100. After blocking in PBS 20% FBS, cells were incubated with primary antibodies during 2 hours at room temperature. The following antibodies and dilutions were used: mouse monoclonal (MAb) anti-troponinT 1/100 (clone JLT-12, Sigma-Aldrich), rabbit polyclonal anti-MuRF1 1/200 (ECM Biosciences, KY, USA) or the 9A12 MAb we developed against DUX4 1/50 [8]. After washing and blocking, cells were incubated during 1 hour at room temperature with Alexa Fluor secondary antibodies 1/100 (goat anti-mouse 488 and anti-rabbit 555, Invitrogen).

### Myotube morphology

Troponin T was stained by immunofluorescence as described above. Myotubes with a width <5 µm were considered «atrophic» and counted from at least 10 random fields. The ratio of atrophic versus total myotubes is expressed in percent as mean ± SD. The significance of the differences between experiments was evaluated with Student's t-test. ***p<0.001 was considered significant.

### Immunodetection on Western blot

Cells were lysed in hypertonic buffer containing 50 mM Tris pH7, 50 mM NaCl, 0.1% NP40, protease inhibitor cocktail (Roche Diagnostics), and 1 mM DTT. For endogenous DUX4 detection, nuclear extracts were prepared with the NE-PER Nuclear and Cytoplasmic Extraction Reagent kit (Thermo Scientific, Rockford, IL, USA) according to the manufacturer. Each cell lysate or nuclear extract was separated by electrophoresis on a 12% polyacrylamide gel in the presence of SDS and transferred to a nitrocellulose membrane (GE Healthcare Europe GmbH, Diegem, Belgium). This Western blot was blocked 1 hour at room temperature with 5% non fat dry milk diluted in phosphate buffered saline (PBS). Membranes were then incubated at 4°C overnight with primary antibodies in PBS 2% BSA. The following antibodies and dilutions were used: 9A12 MAb 1/1000, rabbit polyclonal anti-atrogin1 1/1000 (or anti-MAFbx, ECM Biosciences), anti-GAPDH MAb 1/4000 (Applied Biosystems Ambion), anti-CRYM MAb 1/1000 (or anti-mu-crystallin, Abnova Gmbh, Heidelberg, Germany) and anti-TP53 MAb 1/1000 (Abcam, Cambridge, UK). Membranes were washed in PBS and incubated 1 hour at room temperature with secondary antibodies coupled to horseradish peroxidase (HRP) 1/10000 (GE Healthcare). Proteins were detected on Amersham Hyperfilm ECL (GE Healthcare) with the LiteAbLot (Euroclone, Victoria, Australia), the Lumilight (Roche Diagnostics) or the Super Signal West Femto Maximum Sensitivity Substrate kit (Thermo Scientific). For standardization, the membranes were stripped and immunostaining was performed with either rabbit polyclonal anti-actin serum 1/1000 (Sigma-Aldrich) or anti-TATA Binding Protein MAb 1/2000 (Abcam) as indicated followed by HRP-coupled secondary antibodies 1/10000 (GE Healthcare). A densitometry of the immunoreactive bands was performed with LabImage 1D Software (Kapelan Bio-Imaging). Data are normalized to control loading levels in each sample.

### RNA isolation and 3′RACE

Total RNA was extracted with the NucleoSpin RNA II kit (Macherey-Nagel GmbH, Düren, Germany) as described [8]. Reverse transcription was performed on 1 µg of DNase-treated RNA with the 3′adaptator of the RLM-RACE kit (Applied Biosystems Ambion) and 200 units of SuperScript III reverse transcriptase (Invitrogen) in a 20-µl final volume at 55°C as described [8]. Five µl of the resulting cDNA were amplified by nested PCR in a 50-µl final volume containing 1.25 units of PrimeSTAR HS, 1× GC Buffer (Takara-bio, Japan), and 15 pmol of each primer. The specific outer primer for DUX4 amplification was: 5′-aggcgcaacctctcctagaaac-3′ and the inner primer was: 5′-tggaagcacccctcagcgaggaa-3′. The products were cloned and sequenced to confirm *DUX4* mRNA amplification. For GAPDH cDNA amplification the following primers were used: 5′-gaaggtgaaggtcggagt-3′ and 5′-tgtaaaccatgtagttgaggtc-3′.

## Supporting Information

Figure S1 **CRYM promoter activation by the** ***pCIneo-DUX4*** **expression vector.** (**A**) Schematic representation of the *pCIneo-DUX4* expression vector. It contains the CMV promoter and the full *DUX4* ORF with the *pLAM* region. The DUX4 ORF is represented in black with the two homeobox as in grey. The positions of the different introns are indicated (dark grey boxes). The *pLAM* region encompasses an intron (dark grey box) and the poly-A signal (ATTAAA). (**B**) C2C12 cells were seeded in 6-well plates and co-transfected 24 hours later by Lipofectamin 2000 (Invitrogen) with the CRYM promoter linked to the *firefly* luciferase reporter gene, the internal control phRL-SV40 *renilla* luciferase (Promega), and the *pCIneo-DUX4* expression vector at different concentrations (0, 5, 50 ng/µl). Cells were harvested 16 hours later and processed for enzymatic assays with the Dual Luciferase Assay kit (Promega). Light emissions were recorded on the GlowMax luminometer (Promega), and given in fold activation of firefly versus *renilla* luciferase. Data are presented as mean±SD.
(TIF)

Figure S2 (**A**). PITX1 induce TP53 expression in human myoblasts. Immortalized control myoblasts were transfected with either the *pCIneo–PITX1* expression vector or *pCIneo* as a control. Cells were harvested 24 hours later and total extracts were prepared. Ten µg of proteins were separated by electrophoresis (10% PAGE-SDS) and transferred to a nitrocellulose membrane. Immunodetectection was performed with an anti-TP53 antibody, followed by secondary antibodies coupled to peroxydase (HRP), and revealed with the Lumilight kit (Roche). The protein transfert was confirmed by staining the membrane in Ponceau red that provide a loading control (right panel). The antibodies were then stripped, and the same membrane used for immunodetection with the rabbit antiserum directed against PITX1 (upper pannel). A densitometry of the immunoreactive bands was performed. Data are normalized to actin levels in each sample. The production and the characterisation of this antibody are described in **Fig. S2B**. (**B–C**). Characterisation of the rabbit antiserum directed against PITX1. (**B**) Immortalized control myoblasts were transfected with the *pSMD2-PITX1* expression vector. The cells were lysed 48 hours after transfection, and 40, 20 or 10 µg of protein extracts were separated by electrophoresis (10% PAGE-SDS), and transferred to a nitrocellulose membrane. Immunodetection was performed with a rabbit antiserum directed against two PITX1 specific peptides (Eurogentec), followed by secondary antibodies coupled to peroxydase (HRP), and revealed with the Lumilight kit (Roche). The protein transfer was verified by staining the membrane in Ponceau red that provided a loading control (left panel). Specificity of the antibody against PITX1 was verified by competition with the two immunogenic peptides (pep1, pep2) of the indicated sequences. The PITX1 signal in transfected cells decreased upon competition with a 10-fold excess of one of the two immunogenic peptides and disappeared upon competition with both (Right panel) (**C**) Immortalized control myoblasts were transfected with the indicated vectors. After 48 hours, cells were fixed with 4% PAF. PITX1 (red) was detected by immunofluorescence with the PITX1 rabbit antiserum followed by appropriate secondary antibodies coupled to Alexa Fluor (Invitrogen). The rabbit pre-immune serum was used as a negative control.
(TIF)

Figure S3 Development of siRNA transfection conditions by detection of GAPDH protein in TE671 cells (A) and FSHD primary myoblasts (B). (**A**) We optimized the siRNA transfection conditions with the siPORTNeoFX agent (Ambion) in TE671 cells (human rhabdomyosarcoma cells) using a siRNA targeting GAPDH and a negative control siRNA (nc-siRNA) (provided with the siRNA starter kit, Ambion). The optimal transfection conditions were obtained with the reverse method in which the transfection reagent is introduced into the culture dish before seeding cells. These cells were transfected with GAPDH-siRNA or nc-siRNA and 3 parameters tested: volume of transfection reagent, siRNA concentration and cell density. 72 hours after transfection, 20 µg of protein cell extracts were separated by electrophoresis (12% PAGE-SDS) and transferred onto a nitrocellulose membrane. The protein transfert was confirmed by staining the membrane in Ponceau red. The membrane was then incubated with anti-GAPDH MAb followed by a secondary antibody coupled to peroxidase (HRP) and revealed with the LiteAbLot kit (Euroclone).NT: non-transfected cells. (**B**) 10^5^ cells were seeded in 35 mm culture dish and reverse transfected with GAPDH-siRNA or nc-siRNA (10 nM or 20 nM) and 4 µl of siPORTNeoFX reagent. Cells were harvested 72 hours later and 10 µg of protein extracts were separated by electrophoresis (12% PAGE-SDS) and transferred onto a nitrocellulose membrane. After Ponceau red, staining and rinsing the membrane was incubated with anti-GAPDH MAb followed by secondary antibodies coupled to HRP and revealed with the Lumilight substrate (Roche).
(TIF)

Figure S4 **Evaluation and specificity of siRNA targeting DUX4.** (**A**). TE671 cells were transfected with 10 nM DUX4-siRNA (siRNA1, siRNA2 and siRNA3) or negative control siRNA (nc-siRNA) using reverse transfection (Ambion) and 4 hours later with the *pCIneo-DUX4* vector (DUX4). Three days after transfection the cells were lysed and 20 µg of protein extracts were separated by electrophoresis (12% PAGE-SDS), and transferred to a nitrocellulose membrane. This Western blot was incubated with 9A12 MAb followed by a secondary antibody coupled to peroxidase (HRP) and revealed with the *LiteABlot* kit (Euroclone). NT: non-transfected cells. (**B**). TE671 cells were transfected with 10 nM DUX4-siRNA3 or nc-siRNA using reverse transfection (Ambion) and 4 hours later with the *pCIneo-DUX4* vector (DUX4). The cells were lysed at 24, 48 or 72 hours after the second transfection and 20 µg of protein extracts were analysed by Western blot with 9A12 MAb as above (A). The antibodies were then stripped, and the same membrane revealed with an anti-actin serum (internal control). A densitometry of the immunoreactive bands was performed. Data are normalized to actin levels in each sample. (**C**) TE671 cells were transfected with DUX4c-siRNA or DUX4-siRNA (10 nM) using reverse transfection and 4 hours later with the *pCIneo-DUX4* (DUX4) expression vector as above. The protein extracts were prepared on the third day after *pCIneo* vector transfection and separated by electrophoresis (12% PAGE-SDS), transferred to a Western blot, immunodetected with 9A12 MAb followed by a secondary antibody coupled to peroxidase and revealed with the LiteABlot kit (Euroclone). The antibodies were then stripped, and the same membrane revealed with an anti-actin serum (internal control).
(TIF)

Figure S5 **Determination of AO concentration range to inhibit DUX4 without affecting DUX4c protein expression.** 10^5^ C2C12 cells were seeded per well of a 6-plate dish and co-transfected 24 hours later with 500 ng both *pCIneo-DUX4* and *pCIneo-DUX4c* expression vectors combined with the indicated AOs. The negative control AO mGMCSF3A(−5+20) (nc-AO) targets an unrelated gene transcript in a different species, the murine granulocyte macrophage colony stimulating factor mRNA. The cells were lysed 24 hours after transfection, and 15 µg of protein extracts were separated by electrophoresis (12% PAGE-SDS), and transferred to a nitrocellulose membrane. DUX4 (52-kDa) and DUX4c (47-kDa) were detected on this Western blot with 9A12 MAb followed by secondary antibodies coupled to peroxydase (HRP), and revealed with the Lumilight kit (Roche). After stripping these antibodies, the same membrane was incubated with an anti-actin antibody to provide a loading control. (**A**)The used AO concentration is 150 nM for AOs targeting the *DUX4* mRNA and 600 nM of the nc-AO. (**B–C**): used AO concentrations are indicated. A densitometry of the immunoreactive bands was performed. Data are normalized to actin levels in each sample.
(TIF)

Figure S6 **Endogenous DUX4 protein detection in extracts of human myotubes.** (**A**) 24 hours after seeding immortalized control and FSHD myoblasts were switched to differentiation medium. Cells were harvested 6 days later and a nuclear extract was prepared. 20 µg of nuclear proteins were separated by electrophoresis (12% PAGE-SDS), and transferred onto a nitrocellulose membrane. The protein transfer was confirmed by Ponceau red staining. After rinsing the membrane was incubated with 9A12 MAb followed by secondary antibodies coupled to horseradish peroxidase and revealed with the Femto Super Signal kit (Pierce). (**B–C**) Primary FSHD and control myotubes were harvested 3 days (B) or 4 days (C) after differentiation induction. The protein extracts were prepared, separated by electrophoresis (12% PAGE-SDS) and analysed by Western blot as above. TBP: loading control; TE671 cells transfected with the *pCIneo-DUX4* (DUX4) or the empty *pCIneo* expression vectors (pCI) were used respectively as a positive or negative controls.
(TIF)

Figure S7 **Transfection efficiency on immortal (A–B) or primary (C–D) human skeletal myoblasts.** (**A–B**) Immortal myoblasts were transfected with *pCIneo-EGFP* (A) *or pCIneo-DUX4, -DUX1* (B) expression vectors (NanoJuice, Novagen). (**A**) 48 hours later, nearly 60% of cells expressed EGFP compared with cells counted under bright light (left column). 5 days after differentiation induction cells always expressed EGFP. (**B**) 48 hours after transfection, 10 µg of protein extracts were separated by electrophoresis (12% PAGE-SDS) and transferred onto nitrocellulose membrane. After blocking (5% milk powder), the membrane was incubated with 9A12 MAb followed by a secondary antibody coupled to peroxidase and revealed with the LiteAblot kit (Euroclone). (**C–D**) Primary myoblasts were transfected with *pCIneo-EGFP* (C) *or pCIneo-DUX4, -DUX1* (D) expression vectors (Fugene HD, Roche). (**C**) 24 hours later, nearly 80% of cells expressed EGFP compared with cells counted under bright light (left column). (**D**) 24 hours after transfection, 15 µg of protein extracts were separated by SDS-PAGE electrophoresis (12%) and analysed by Western blot as above.
(TIF)
