# Supplementary figures and images for: The FSHD Atrophic Myotube Phenotype Is Caused by DUX4 Expression
Source: PLoS One. 2011 Oct 28;6(10):e26820. doi: 10.1371/journal.pone.0026820 (PMC3203905; doi:10.1371/journal.pone.0026820)

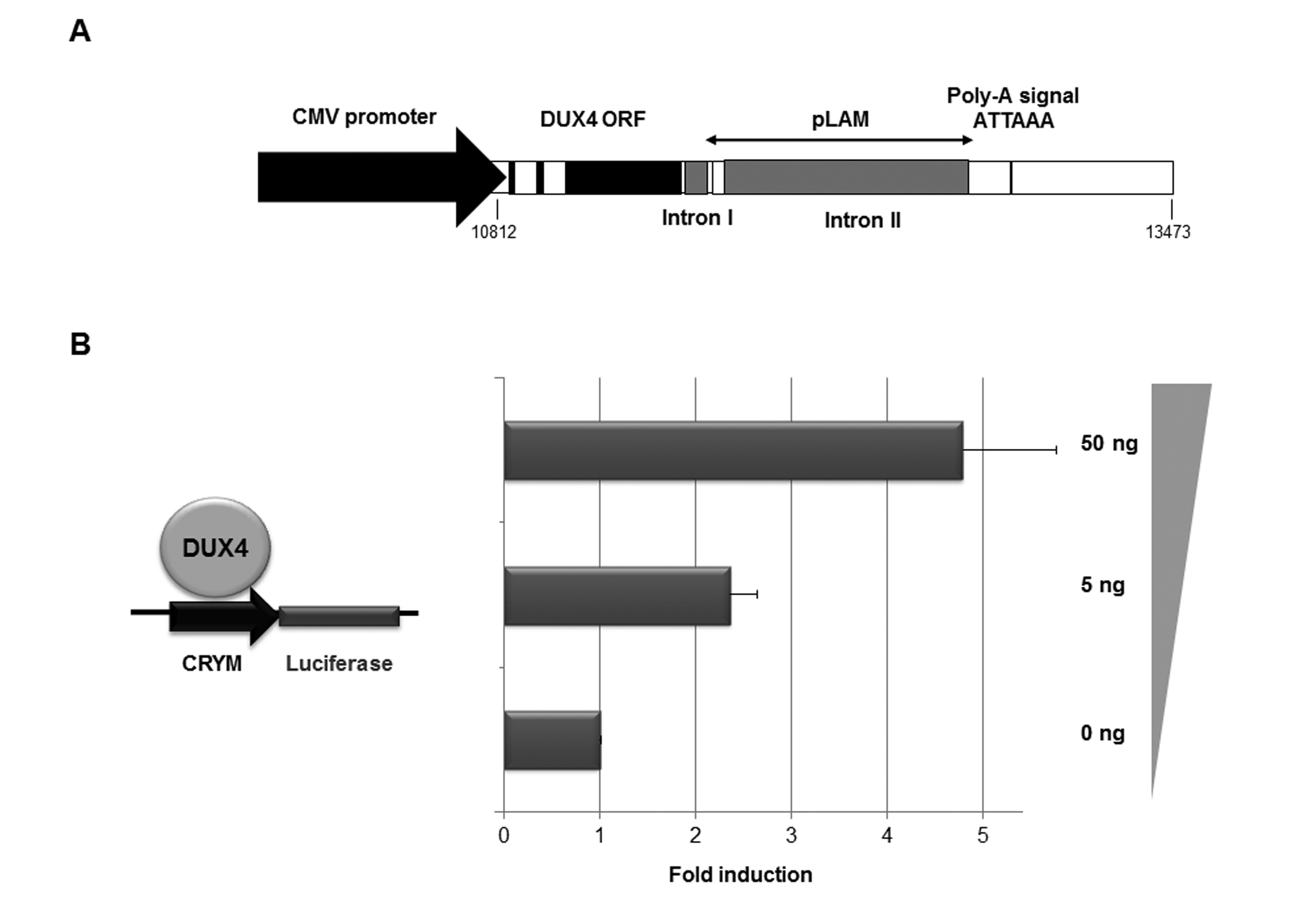

Supplement: Figure S1 — CRYM promoter activation by the pCIneo-DUX4 expression vector. (A) Schematic representation of the pCIneo-DUX4 expression vector. It contains the CMV promoter and the full DUX4 ORF with the pLAM region. The DUX4 ORF is represented in black with the two homeobox as in grey. The positions of the different introns are indicated (dark grey boxes). The pLAM region encompasses an intron (dark grey box) and the poly-A signal (ATTAAA). (B) C2C12 cells were seeded in 6-well plates and co-transfected 24 hours later by Lipofectamin 2000 (Invitrogen) with the CRYM promoter linked to the firefly luciferase reporter gene, the internal control phRL-SV40 renilla luciferase (Promega), and the pCIneo-DUX4 expression vector at different concentrations (0, 5, 50 ng/µl). Cells were harvested 16 hours later and processed for enzymatic assays with the Dual Luciferase Assay kit (Promega). Light emissions were recorded on the GlowMax luminometer (Promega), and given in fold activation of firefly versus renilla luciferase. Data are presented as mean±SD. (TIF) [file pone.0026820.s001.tif]

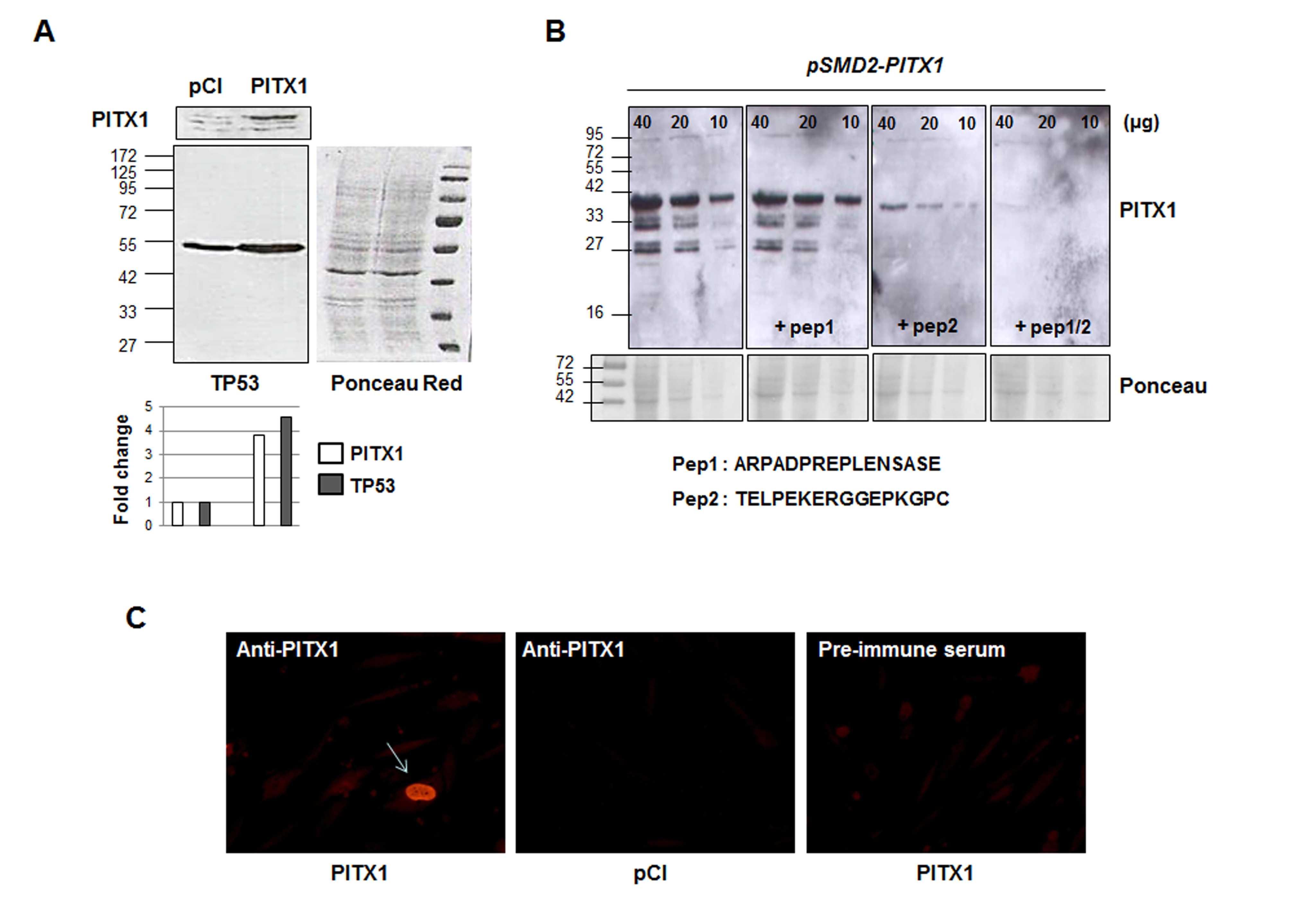

Supplement: Figure S2 — (A). PITX1 induce TP53 expression in human myoblasts. Immortalized control myoblasts were transfected with either the pCIneo–PITX1 expression vector or pCIneo as a control. Cells were harvested 24 hours later and total extracts were prepared. Ten µg of proteins were separated by electrophoresis (10% PAGE-SDS) and transferred to a nitrocellulose membrane. Immunodetectection was performed with an anti-TP53 antibody, followed by secondary antibodies coupled to peroxydase (HRP), and revealed with the Lumilight kit (Roche). The protein transfert was confirmed by staining the membrane in Ponceau red that provide a loading control (right panel). The antibodies were then stripped, and the same membrane used for immunodetection with the rabbit antiserum directed against PITX1 (upper pannel). A densitometry of the immunoreactive bands was performed. Data are normalized to actin levels in each sample. The production and the characterisation of this antibody are described in Fig. S2B. (B–C). Characterisation of the rabbit antiserum directed against PITX1. (B) Immortalized control myoblasts were transfected with the pSMD2-PITX1 expression vector. The cells were lysed 48 hours after transfection, and 40, 20 or 10 µg of protein extracts were separated by electrophoresis (10% PAGE-SDS), and transferred to a nitrocellulose membrane. Immunodetection was performed with a rabbit antiserum directed against two PITX1 specific peptides (Eurogentec), followed by secondary antibodies coupled to peroxydase (HRP), and revealed with the Lumilight kit (Roche). The protein transfer was verified by staining the membrane in Ponceau red that provided a loading control (left panel). Specificity of the antibody against PITX1 was verified by competition with the two immunogenic peptides (pep1, pep2) of the indicated sequences. The PITX1 signal in transfected cells decreased upon competition with a 10-fold excess of one of the two immunogenic peptides and disappeared upon competition with both (Right p [file pone.0026820.s002.tif]

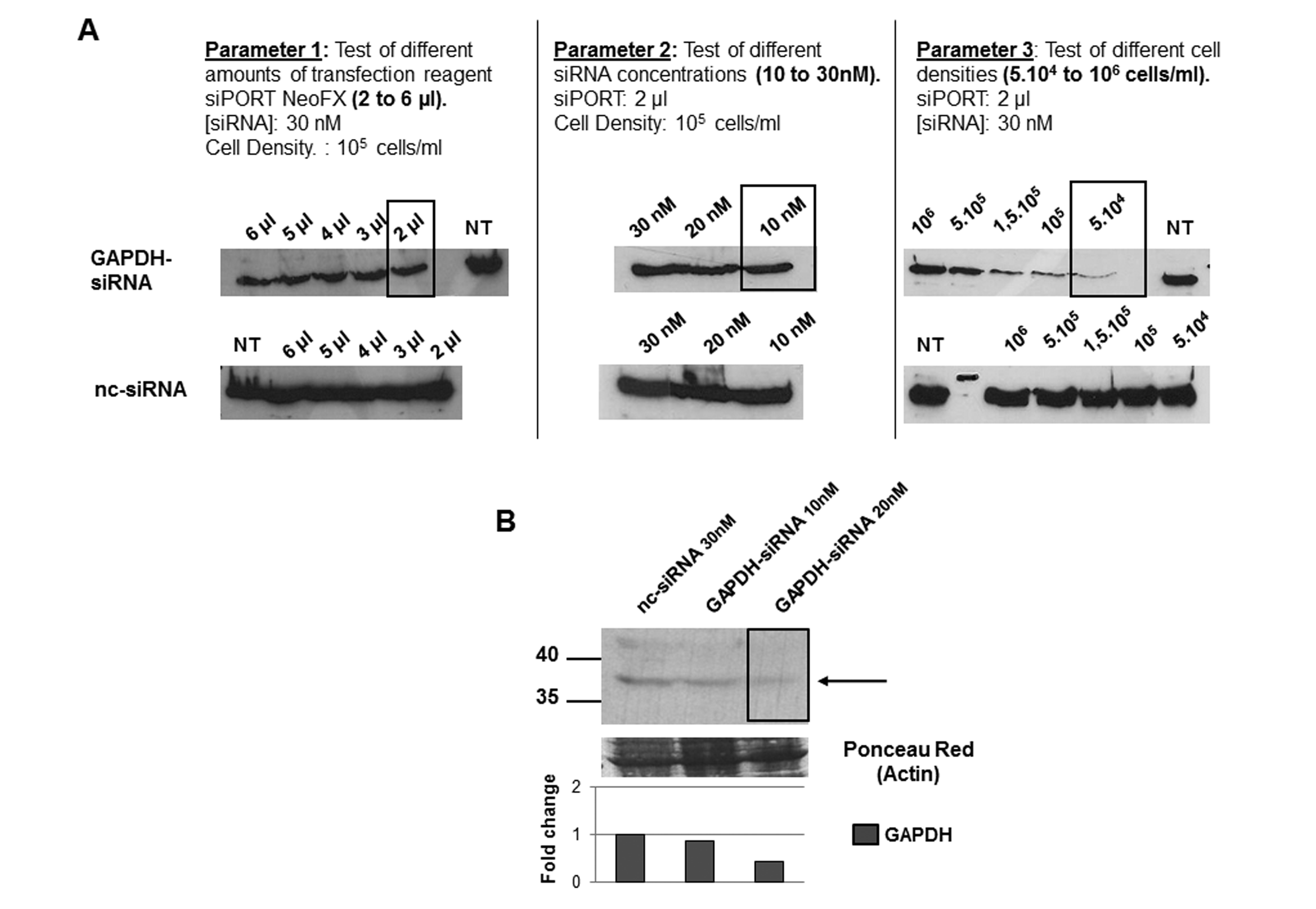

Supplement: Figure S3 — Development of siRNA transfection conditions by detection of GAPDH protein in TE671 cells (A) and FSHD primary myoblasts (B). (A) We optimized the siRNA transfection conditions with the siPORTNeoFX agent (Ambion) in TE671 cells (human rhabdomyosarcoma cells) using a siRNA targeting GAPDH and a negative control siRNA (nc-siRNA) (provided with the siRNA starter kit, Ambion). The optimal transfection conditions were obtained with the reverse method in which the transfection reagent is introduced into the culture dish before seeding cells. These cells were transfected with GAPDH-siRNA or nc-siRNA and 3 parameters tested: volume of transfection reagent, siRNA concentration and cell density. 72 hours after transfection, 20 µg of protein cell extracts were separated by electrophoresis (12% PAGE-SDS) and transferred onto a nitrocellulose membrane. The protein transfert was confirmed by staining the membrane in Ponceau red. The membrane was then incubated with anti-GAPDH MAb followed by a secondary antibody coupled to peroxidase (HRP) and revealed with the LiteAbLot kit (Euroclone).NT: non-transfected cells. (B) 105 cells were seeded in 35 mm culture dish and reverse transfected with GAPDH-siRNA or nc-siRNA (10 nM or 20 nM) and 4 µl of siPORTNeoFX reagent. Cells were harvested 72 hours later and 10 µg of protein extracts were separated by electrophoresis (12% PAGE-SDS) and transferred onto a nitrocellulose membrane. After Ponceau red, staining and rinsing the membrane was incubated with anti-GAPDH MAb followed by secondary antibodies coupled to HRP and revealed with the Lumilight substrate (Roche). (TIF) [file pone.0026820.s003.tif]

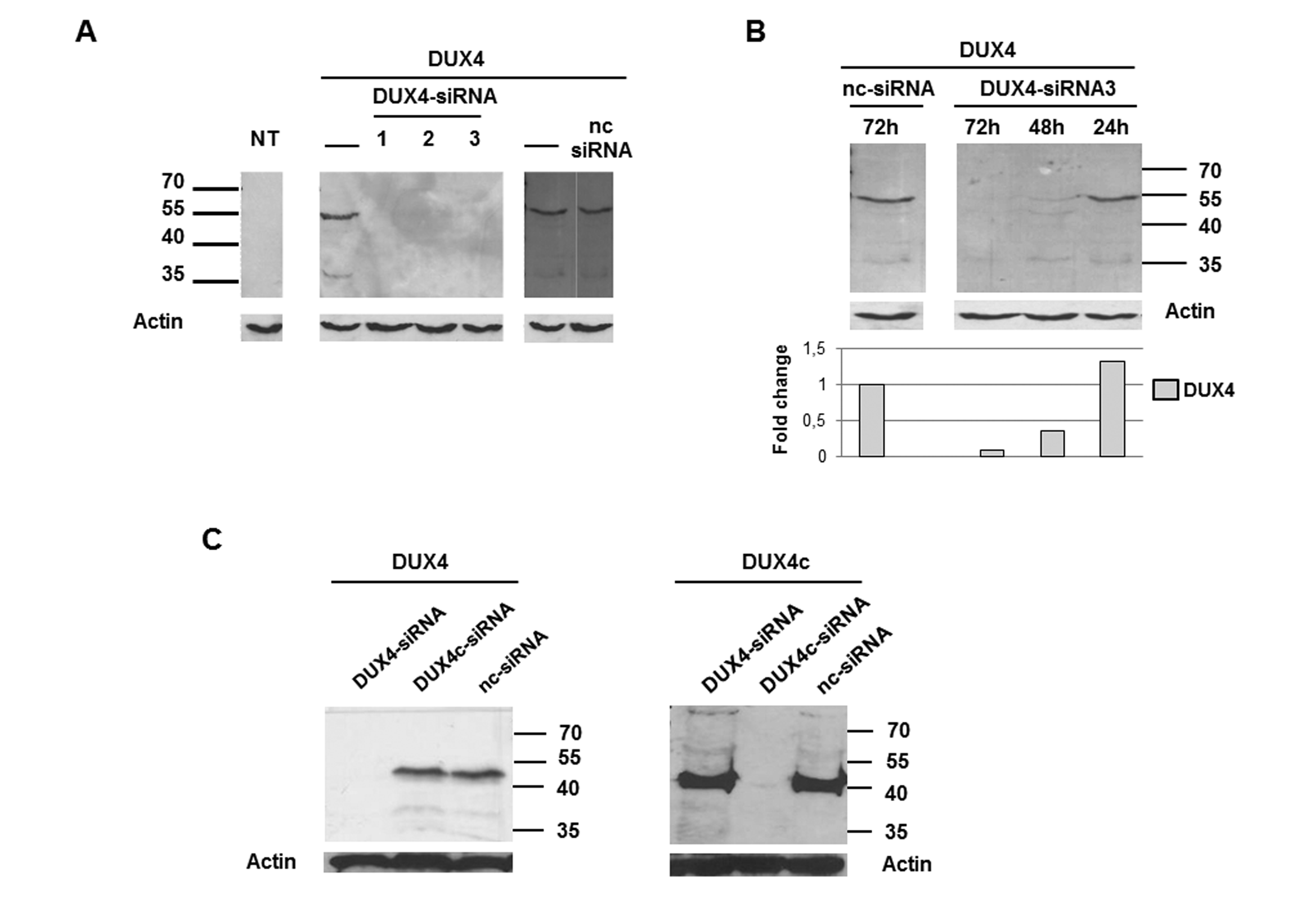

Supplement: Figure S4 — Evaluation and specificity of siRNA targeting DUX4. (A). TE671 cells were transfected with 10 nM DUX4-siRNA (siRNA1, siRNA2 and siRNA3) or negative control siRNA (nc-siRNA) using reverse transfection (Ambion) and 4 hours later with the pCIneo-DUX4 vector (DUX4). Three days after transfection the cells were lysed and 20 µg of protein extracts were separated by electrophoresis (12% PAGE-SDS), and transferred to a nitrocellulose membrane. This Western blot was incubated with 9A12 MAb followed by a secondary antibody coupled to peroxidase (HRP) and revealed with the LiteABlot kit (Euroclone). NT: non-transfected cells. (B). TE671 cells were transfected with 10 nM DUX4-siRNA3 or nc-siRNA using reverse transfection (Ambion) and 4 hours later with the pCIneo-DUX4 vector (DUX4). The cells were lysed at 24, 48 or 72 hours after the second transfection and 20 µg of protein extracts were analysed by Western blot with 9A12 MAb as above (A). The antibodies were then stripped, and the same membrane revealed with an anti-actin serum (internal control). A densitometry of the immunoreactive bands was performed. Data are normalized to actin levels in each sample. (C) TE671 cells were transfected with DUX4c-siRNA or DUX4-siRNA (10 nM) using reverse transfection and 4 hours later with the pCIneo-DUX4 (DUX4) expression vector as above. The protein extracts were prepared on the third day after pCIneo vector transfection and separated by electrophoresis (12% PAGE-SDS), transferred to a Western blot, immunodetected with 9A12 MAb followed by a secondary antibody coupled to peroxidase and revealed with the LiteABlot kit (Euroclone). The antibodies were then stripped, and the same membrane revealed with an anti-actin serum (internal control). (TIF) [file pone.0026820.s004.tif]

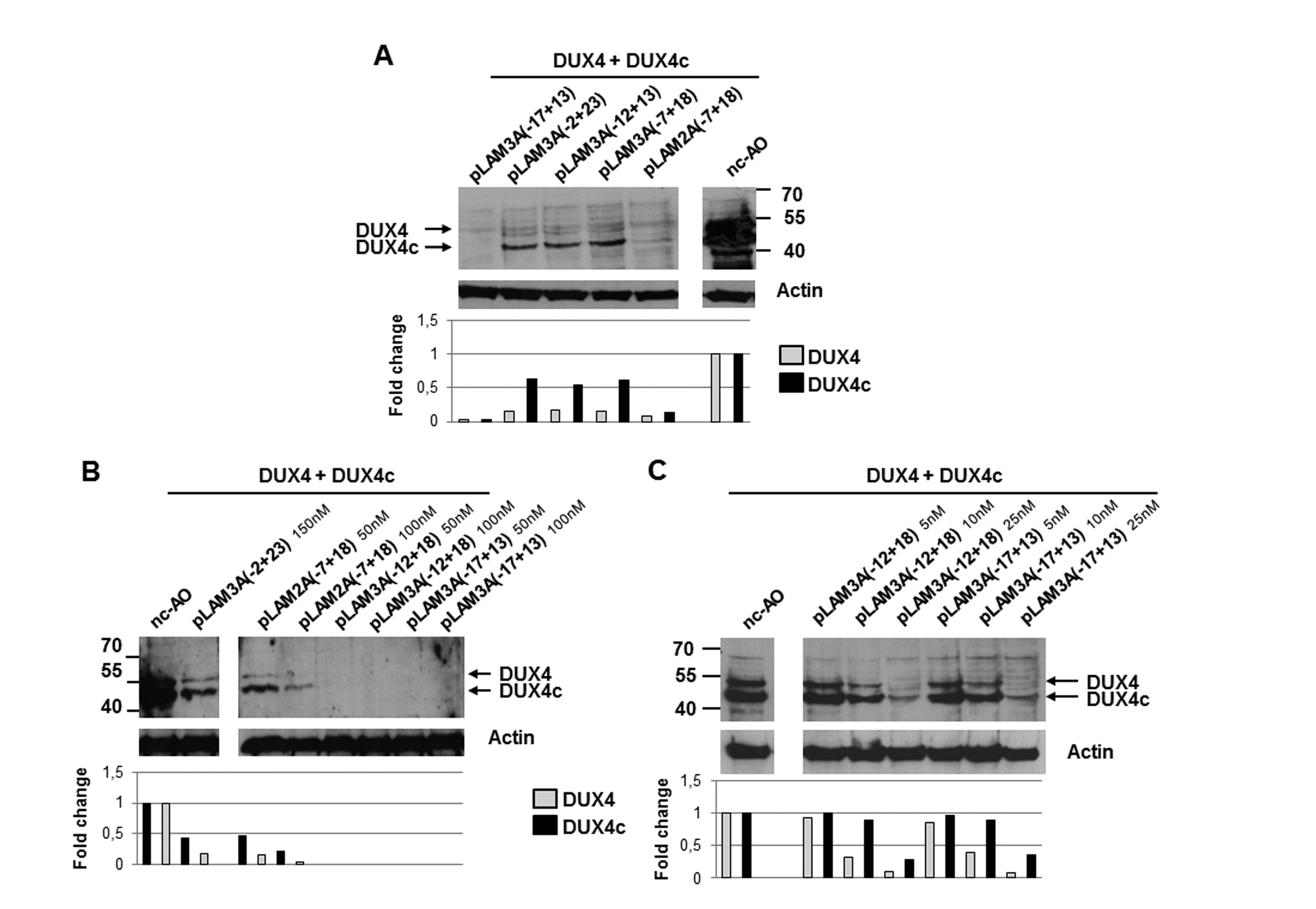

Supplement: Figure S5 — Determination of AO concentration range to inhibit DUX4 without affecting DUX4c protein expression. 105 C2C12 cells were seeded per well of a 6-plate dish and co-transfected 24 hours later with 500 ng both pCIneo-DUX4 and pCIneo-DUX4c expression vectors combined with the indicated AOs. The negative control AO mGMCSF3A(−5+20) (nc-AO) targets an unrelated gene transcript in a different species, the murine granulocyte macrophage colony stimulating factor mRNA. The cells were lysed 24 hours after transfection, and 15 µg of protein extracts were separated by electrophoresis (12% PAGE-SDS), and transferred to a nitrocellulose membrane. DUX4 (52-kDa) and DUX4c (47-kDa) were detected on this Western blot with 9A12 MAb followed by secondary antibodies coupled to peroxydase (HRP), and revealed with the Lumilight kit (Roche). After stripping these antibodies, the same membrane was incubated with an anti-actin antibody to provide a loading control. (A)The used AO concentration is 150 nM for AOs targeting the DUX4 mRNA and 600 nM of the nc-AO. (B–C): used AO concentrations are indicated. A densitometry of the immunoreactive bands was performed. Data are normalized to actin levels in each sample. (TIF) [file pone.0026820.s005.tif]

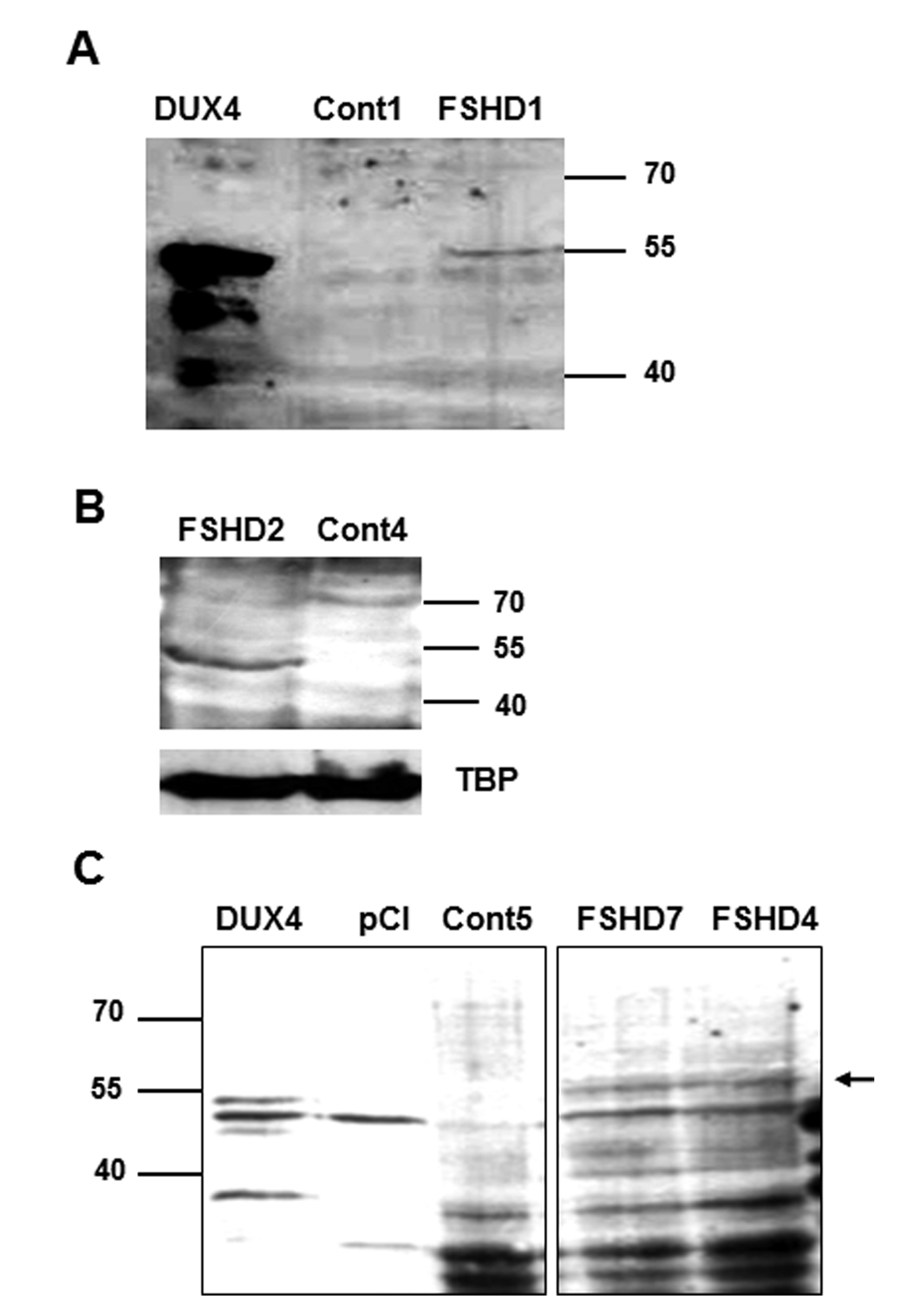

Supplement: Figure S6 — Endogenous DUX4 protein detection in extracts of human myotubes. (A) 24 hours after seeding immortalized control and FSHD myoblasts were switched to differentiation medium. Cells were harvested 6 days later and a nuclear extract was prepared. 20 µg of nuclear proteins were separated by electrophoresis (12% PAGE-SDS), and transferred onto a nitrocellulose membrane. The protein transfer was confirmed by Ponceau red staining. After rinsing the membrane was incubated with 9A12 MAb followed by secondary antibodies coupled to horseradish peroxidase and revealed with the Femto Super Signal kit (Pierce). (B–C) Primary FSHD and control myotubes were harvested 3 days (B) or 4 days (C) after differentiation induction. The protein extracts were prepared, separated by electrophoresis (12% PAGE-SDS) and analysed by Western blot as above. TBP: loading control; TE671 cells transfected with the pCIneo-DUX4 (DUX4) or the empty pCIneo expression vectors (pCI) were used respectively as a positive or negative controls. (TIF) [file pone.0026820.s006.tif]

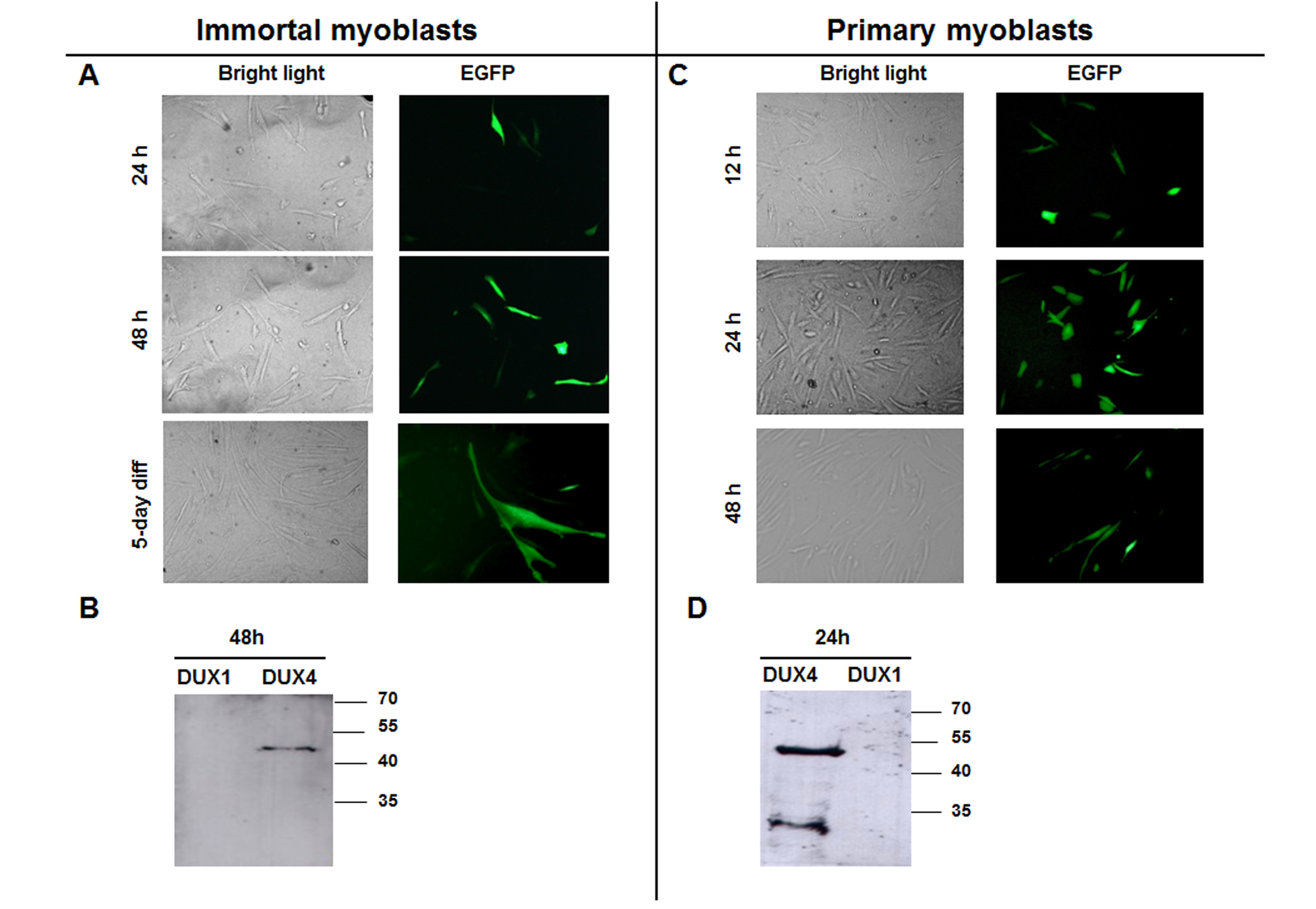

Supplement: Figure S7 — Transfection efficiency on immortal (A–B) or primary (C–D) human skeletal myoblasts. (A–B) Immortal myoblasts were transfected with pCIneo-EGFP (A) or pCIneo-DUX4, -DUX1 (B) expression vectors (NanoJuice, Novagen). (A) 48 hours later, nearly 60% of cells expressed EGFP compared with cells counted under bright light (left column). 5 days after differentiation induction cells always expressed EGFP. (B) 48 hours after transfection, 10 µg of protein extracts were separated by electrophoresis (12% PAGE-SDS) and transferred onto nitrocellulose membrane. After blocking (5% milk powder), the membrane was incubated with 9A12 MAb followed by a secondary antibody coupled to peroxidase and revealed with the LiteAblot kit (Euroclone). (C–D) Primary myoblasts were transfected with pCIneo-EGFP (C) or pCIneo-DUX4, -DUX1 (D) expression vectors (Fugene HD, Roche). (C) 24 hours later, nearly 80% of cells expressed EGFP compared with cells counted under bright light (left column). (D) 24 hours after transfection, 15 µg of protein extracts were separated by SDS-PAGE electrophoresis (12%) and analysed by Western blot as above. (TIF) [file pone.0026820.s007.tif]
